# Vaginal chlorhexidine gluconate versus fluconazole for recurrent vulvovaginal candidiasis: A randomized noninferiority trial

**DOI:** 10.1371/journal.pone.0340862

**Published:** 2026-01-20

**Authors:** Cathrin Alvendal, Tyra Hasselrot, Margaux Balduck, Gabriella Edfeldt, Ina Schuppe-Koistinen, Annelie Brauner, Daniel Altman, Kristina Broliden, Nina Bohm-Starke

**Affiliations:** 1 Department of Clinical Sciences, Division of Obstetrics and Gynecology, Karolinska Institutet, Danderyd Hospital, Stockholm, Sweden; 2 Department of Medicine Solna, Division of Infectious Diseases, Karolinska Institutet, Department of Infectious Diseases, Karolinska University Hospital, Center for Molecular Medicine, Stockholm, Sweden; 3 Centre for Translational Microbiome Research, Department of Microbiology, Tumor and Cell Biology, Karolinska Institutet, Stockholm, Sweden; 4 Department of Microbiology, Tumor and Cell Biology, Division of Clinical Microbiology, Karolinska Institutet and Karolinska University Hospital, Stockholm, Sweden; 5 Department of Women’s and Children’s Health, Uppsala University, Uppsala, Sweden; Universidade dos Açores Departamento de Biologia: Universidade dos Acores Departamento de Biologia, PORTUGAL

## Abstract

**Background:**

Recurrent vulvovaginal candidiasis (RVVC) is a common condition characterized by frequent relapses, often without a clearly identifiable cause. Fluconazole (FLZ) is the standard treatment but concerns about emerging resistance and drug interaction highlight the need for alternative therapies. Chlorhexidine gluconate (CHG), known for its antifungal and biofilm-disrupting properties, has been proposed as a potential alternative.

**Objective:**

To evaluate the efficacy, tolerability, and microbiome impact of a vaginal CHG formulation compared to oral FLZ in the treatment of RVVC caused by *Candida albicans*.

**Methods:**

An open label randomized non-inferiority trial was conducted to compare vaginal CHG and FLZ treatments. Primary outcome was negative cultures for *C. albicans*. Resistance profiles and changes in the vaginal microbiome composition were also assessed.

**Results:**

The study was terminated early due to local irritation associated with CHG and the study design was transitioned into a pilot study. CHG treatment showed comparable efficacy to FLZ in clearing *C. albicans* infections and preventing recurrences, although the sample size was limited. All 11 participants in the FLZ group cleared the infection after one week treatment, compared to 9 out 11 in the CHG group. No harmful changes to the vaginal microbiome were observed in the CGH or FLZ group. FLZ promoted a shift toward *Lactobacillus crispatus* dominance, unlike CHG. Notably, 16% of *C. albicans* isolates exhibited reduced susceptibility or resistance to FLZ.

**Conclusion:**

Due to the limited number of participants, we cannot conclusively determine whether CHG is non-inferior to FLZ in terms of efficacy for clearing acute C. *albicans* infections or preventing recurrences. While the current CHG formulation caused local irritation and is not suitable for clinical use, its antifungal and biofilm-inhibiting properties remain promising. Further development of less irritative CHG formulations may offer a valuable alternative for RVVC treatment, particularly in the context of rising FLZ resistance.

## Introduction

Vulvovaginal candidiasis (VVC) is a common fungal infection that predominantly affects women of childbearing age. The most prevalent causative agent is *Candida albicans* (*C. albicans*), responsible for approximately 90% of cases, followed by *C. glabrata*, *C. parapsilosis,* and *C. tropicalis* [1,2]. VVC affects an estimated 70–75% of women at some point in their lives, with known risk factors including diabetes mellitus, pregnancy, and antibiotic use [2–4]. Relapses are frequent, and 5–9% of women experience recurrent vulvovaginal candidiasis (RVVC), defined as three or more episodes per year [2,5–7].

The underlying causes of RVVC are unclear and the patients are in most cases healthy without obvious risk factors. Theories of underlying causes to RVVC are alterations of the vaginal immune system with increased susceptibility in the host, specific virulent characteristics of the involved *Candida* strains and genetic polymorphisms [5,7]. Adhesion to epithelial surfaces is the first essential steps towards colonization and infections of Candida spp [1]. Another important fungal virulence factor is the formation of biofilm, correlating to resistance to antifungals [8–10].

The standard treatment for RVVC involves long-term administration of oral fluconazole (FLZ) [7]. While many patients tolerate FLZ well, adverse effects have been reported, and long-term use is not suitable for all individuals. Notably, FLZ interacts with several commonly used medications in this patient population, with the most serious concern being QT interval prolongation and cardiac arrhythmia [11,12]. Furthermore, FLZ is contraindicated during pregnancy, a condition that itself increases susceptibility to VVC [13,14].

As an alternative to systemic antifungal agents, broad-spectrum antiseptics such as chlorhexidine gluconate (CHG) could be used. In our *in vitro* studies, we demonstrated that *C. albicans* forms biofilms, a process inhibited by CHG. Additionally, CHG exhibited direct fungicidal activity against *C*. *albicans* [15]. Clinically, CHG is used in the treatment of oral candidiasis [16,17]. In Sweden, CHG-based vaginal creams are employed during gynecological examinations, particularly in obstetric surgical settings [18]. However, a potential concern with antiseptic use is the disruption of the cervicovaginal microbiome. Currently, there is limited data on how local CHG application affects the composition and stability of vaginal microbiota. The overall aim of this study was to evaluate whether vaginally applied CHG could serve as an effective alternative to oral FLZ for both acute treatment and prophylaxis of RVVC.

## Materials and methods

### Study design and participants

This study was planned as a Phase II, open label, randomized, controlled, non-inferiority trial designed to evaluate whether a 1% CHG vaginal cream (n = 30) is non-inferior to standard oral FLZ treatment (n = 30) in women diagnosed with RVVC at the Women’s Department at Danderyd Hospital, Stockholm, Sweden. FLZ served as active control. The hypothesis was that CHG was non-inferior to FLZ in the efficacy to treat an acute episode of RVVC and to prevent recurrences. As it turned out, the trial ended prematurely due to unexpected adverse events in the CHG group, and the study design changed to a pilot study. However, we chose to report the trial in accordance with the CONSORT Statement extension for non-inferiority and equivalent trials [19,20]. It is registered at EduraCT (no: 2020-000758-81) and Clinical.Trials.gov. (Identifier NCT05059145). The first ethical approval on the clinical trial was granted by the Swedish Ethical Review Authority on September 8, 2020 (Dnr 2020−04035) and the second application including more advanced molecular analyses was granted on February 6, 2022 (Dnr 2021-06538-02). All participants provided written informed consent prior to enrollment, witnessed by the responsible investigator for each participant.

The primary objective was to determine whether vaginal application of CHG is non-inferior to oral FLZ for both acute and prophylactic treatment of RVVC caused by *C. albicans*. The primary outcome was negative *C. albicans* cultures one week after completing acute treatment. Secondary outcomes included: 1) Negative *C. albicans* cultures after three months of prophylactic treatment and an additional three-month observation period, 2) Evaluation of clinical symptoms and findings at all follow-up visits, 3) Number of relapses during the prophylactic and observation phase, 4) Incidence and nature of adverse events, and 5) Impact of treatment on the composition of the vaginal microbiome.

Inclusion criteria were: women aged 18–50 years with a history of RVVC, defined as ≥ 3 episodes of Candida infection in the past year; current symptoms consistent with acute vulvovaginal candidiasis; and culture-confirmed *C. albicans* susceptible to FLZ. Exclusion criteria included: severe somatic or psychiatric illness; use of immunosuppressive medication; pregnancy or lactation; presence of other ongoing gynecological infections; vaginal intercourse within the past 24 hours; known allergy to FLZ or CHG; and use of escitalopram or other medications known to affect the QT interval.

**Fig 1:**
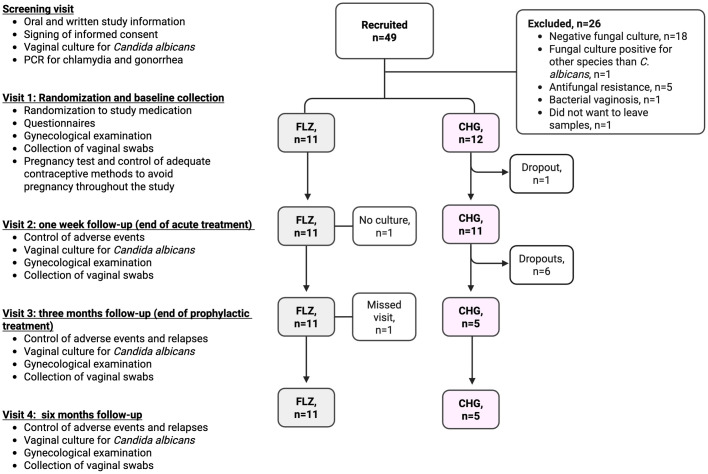
Flow-chart of inclusion and exclusion of study participants.

The study consisted of five scheduled visits over a six-month period (**see flow-chart, Fig 1**). Recruitment was conducted via advertisements at regional gynecological clinics and on social media, inviting women with RVVC to contact the research midwife at the Women’s Department at Danderyd Hospital for further information. Women meeting the age and symptom criteria were invited to a screening visit. At this visit, participants received both oral and written information about the study from the research midwife and provided informed consent. Vaginal samples were collected for fungal culture and PCR testing for *Chlamydia trachomatis* and *Neisseria gonorrhoeae*. Final inclusion and randomization occurred approximately two days later, contingent upon confirmation of *C. albicans* infection by culture. Participants were then scheduled for Visit 1, during which they underwent a clinical examination and were randomized to receive the study medication. At this visit, participants also completed a web-based study specific questionnaire covering medical and reproductive history.

Participants were randomized to one of two treatment arms: a) The CHG group received Hibitane® vaginal cream, 7.5 mL nightly for 7 days as acute treatment, followed by prophylactic treatment with 7.5 mL once weekly for 11 consecutive weeks, b) The FLZ group received oral Fluconazole® 150 mg every third day for the first three doses (acute phase), followed by 150 mg once weekly for 11 consecutive weeks (prophylactic phase).

The randomization ratio between the treatment groups was 1:1. A computer generated block randomization of 15 participants in each block was performed and allocation to the study medications was carried out via opening of opaque sealed envelopes in a consecutive order by the research midwife. All participants were identified through a patient log with name and Swedish personal identification number and the randomization number. The treatment was not blinded to participants or investigators due to the difference in appearance and application of the study drugs.

**Fig 2:**
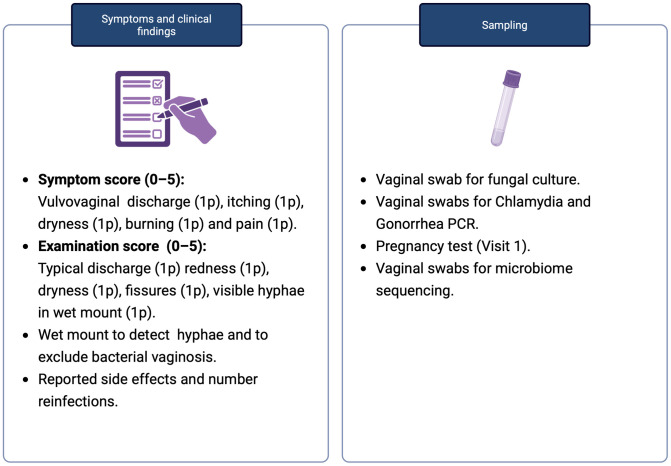
Findings and samples documented and collected at each study visit. Symptom score was based on self-reported symptoms by participants. Examination score was evaluated by the gynaecologist at the gynaecological examination.

Visit 2 was scheduled after completion of the one-week acute treatment phase. At this visit, participants underwent a clinical examination and sampling, including a vaginal culture for *C. albicans* which served as the primary outcome measure. Following Visit 2, participants entered a three-month prophylactic treatment phase, which concluded with Visit 3. The final phase of the study consisted of a three-month observation period to monitor recurrences. This phase concluded with a follow-up visit at six months post-baseline. Details of the study timeline, procedures, and sampling at each visit are illustrated in **Figs 1 and 2**. A weekly web-based electronic diary system (Entermedic, Entergate AB, Halmstad, Sweden) was used to monitor treatment complications and adverse events. Participants received a personalized link via email each week during both the acute and prophylactic treatment phases, allowing them to complete the diary online. During the observational phase, participants were instructed to report any suspected relapses and to contact the research midwife or study investigators. To facilitate early detection of recurrences, participants were provided with self-sampling kits for vaginal fungal cultures, which could be used at home. In cases where *C. albicans* was confirmed by culture, participants were offered the same treatment regimen used during the study, provided it had been effective and well-tolerated. If adverse events had occurred or the previous treatment was ineffective, an individualized treatment plan was implemented. The full study protocol is included in Supporting information (S1 File).

### Candida cultures

Vaginal swabs were transported in liquid amies transport medium and cultured on CHROMagar Candida Plus and incubated at 37°C for 24-48h. C. *albicans* typically appears as green colonies. To confirm species identification, MALDI-TOF was done. Antifungal susceptibility testing was performed using YeastOne Sensititre (Thermo Fisher), according to the manufacturer´s recommendation. When MIC-values were classified as I or R, verification was performed using the European Committee for Antimicrobial Susceptibility Testing (EUCAST) broth microdilution method [21].

### Microbiome sequencing

a)
***Microbiome sample collection***

One vaginal swab for microbiome analysis (FLOQSwabs™, Copan Flock Technologies, Brescia, Italy) was collected at each visit. After sampling, the swab was transferred to 0.8 ml DNA/RNA-shield (Zymo Research, Irvine, CA, USA) containing FluidX tubes (Brooks Life Sciences, Chelmsford, MA, USA), according to previously described practice [22].

b)
***DNA extraction and sequencing***

DNA extraction was performed as previously reported [22]. In short, Quant-iT dsDNA Assay kits (Invitrogen, ThermoFisher, USA) were applied for DNA quantification, followed by normalization to 50 ng. DNA libraries were prepared using MGIEasy FS DNA Library Prep Set kit (MGI, Shenzhen, China). Employed libraries were assessed using Quant-iT dsDNA Assay kits and TapeStation D1000 kit (Agilent, USA) to measure their concentration and fragment size, respectively. In addition, all were pooled with equal amounts of sample DNA. Using a DNBSEQ-T7 sequencer (MGI, Shenzhen, China) 100 bp paired-end sequencing was performed on circularized DNA libraries. Additionally, for both the extraction and sequencing step, positive (ZymoBIOMICS Community standard, Zymo Research, Irvine, CA, USA) and negative (DNA/RNA shield) controls were included.

c)
***Taxonomic annotation***

Analysis and annotation of shotgun libraries were performed using the Snakemake workflow (ctmrbio/stag-mwc: StaG v0.7.0) [23]. By mapping against the human genome version GRCh38 using Kraken2 (v2.0.8-beta), human DNA was identified. This was then removed, followed by taxonomic annotation by MetaPhlAn 4.0.6 [24].

All samples reached 20 M total reads. Taxa were included if they exceeded a 0.005% relative abundance in a minimum of 6% of the samples, or at least 5% relative abundance in no fewer than 2% of samples. This allowed for filtering of very rare taxa while maintaining biologically relevant low abundant taxa and retaining taxa with a relatively high abundance in a smaller number of samples. Applying both thresholds removes unwanted noise but keeps variation. Additionally, by filtering out samples that did not reach 60% total relative abundance, 1 sample collected from a woman using CHG at the 1-week timepoint, was excluded from further analysis. After filtering, relative abundances were re-normalized to 100%.

d)
***Microbiome data analysis***

All microbiome analyses were performed in R version v4.4.1 [25]. Based on relative abundances the most abundantly present species was identified per patient at each individual timepoint. This major species shift was visualized in an alluvial plot using ggplot2 v3.5.1 and the ggalluvial package v0.12.5 [26–28].

### Statistical analyses

The sample size and power calculation for the primary outcome (negative *C. albicans* culture following treatment) was based on data from Alvendal et al. [29]. In that study, 100% of participants had positive *C. albicans* cultures prior to standard FLZ treatment for RVVC, and 14% remained culture-positive post-treatment, corresponding to an 86% success rate. Assuming a significance level (α) of 5%, a power (1-β) of 90%, and a non-inferiority margin of 30%, the current study was designed to demonstrate that the experimental treatment with CHG is not inferior to standard FLZ treatment. With an expected success rate of 86% in both groups, a total of 46 participants (23 per group) was required to achieve 90% power to exclude a difference in effect-size greater than 30%.

Clinical and sociodemographic characteristics were compared between the two treatment groups using data from the screening visit questionnaires. The Mann-Whitney U-test was used for continuous variables, and Fischer’s exact test was applied for categorical variables. A p-value < 0.05 was considered statistically significant. Details of the microbiome data analysis are provided above.

## Results

### Study participants

Study recruitment began April 27, 2022, and the final study visit was completed November 27, 2024. Although many women expressed interest and contacted the research midwife, only 49 met the eligibility criteria and were invited for a screening visit. Of these, 26 were excluded due to not fulfilling the inclusion criteria. The remaining 23 participants were randomized to receive either CHG (n = 12) or FLZ (n = 11) (Fig 1). Among the 32 women with positive vaginal cultures for *Candida* species at screening, one was colonized with *C*. *parapsilosis,* and five (16%) had *C. albicans* isolates with reduced susceptibility to FLZ (minimum inhibitory concentration [MIC] ≥ 4 mg/L).

The median age of participants was 32 years (range: 23–46), and the median duration of RVVC was 6 years (range: 1–25). All participants reported being in good general health. No statistically significant differences were observed between the CHG and FLZ groups in any of the baseline clinical or sociodemographic characteristics, as assessed by the screening questionnaires (Table 1).

**Table 1:** Clinical characteristics of the study participants.

	CHG (n = 12)	FLZ (n = 11)	*p-value* ^†^
	Median or number^†^ (range or %)		
Age (years)	33 (24–46)	32 (23–45)	0.89
BMI (kg/m^2^)	22 (20–27)	22 (19–26)	0.51
Smoking	0 (0)	0 (0)	>0.99
Stable partner	11 (100	12 (100)	>0.99
Regular menstruations	7 (58)	8 (73)	>0.99
No. of pregnancies	1 (0–6)	2 (0–4)	0.46
No. of children	0 (0–3)	1 (0–3)	0.82
**Contraceptive use**			
Hormonal IUD	5 (42)	3 (27)	0.30
Birth control pills	4 (33)	2 (18)	>0.99
No contraceptive	3 (25)	6 (55)	0.31
**Previous gynecological infections**			
Chlamydia	1 (8)	3 (27)	0.32
Herpes	0 (0)	1 (9)	0.48
Condyloma	5 (42)	1 (9)	0.16
Foul-smelling vaginal discharge	1 (8)	2 (18)	0.59
**RVVC**			
Duration of RVVC (years)	6 (2 − 25)	7 (1 − 11)	0.50
Vaginal yeast infections at least once a month	9 (75)	9 (83)	>0.99

Based on self-report through questionnaires filled out at the screening visit. ^†^A Mann-Whitney U-test was applied to compare continuous variables between the study groups, and Fischer’s exact test was applied to compare binary variables. Abbreviations: CHG = chlorhexidine gluconate, FLZ = fluconazole, BMI = body mass index, no. = number, IUD = intrauterine device.

From the outset of the study, 8 out of 12 participants in the CHG group reported adverse events (AEs), most commonly described as painful vulvovaginal burning following application of the vaginal cream. Although these AEs were not classified as serious, typically resolving within a few hours to 1–2 days, they were frequent and uncomfortable. In contrast, 3 out of 11 participants in the FLZ group reported fewer and non-serious AEs, with nausea, headache, and diarrhea occurring in a minority of cases. A comprehensive summary of all reported AEs is provided in S1 and S2 Tables.

Despite the non-serious classification of AEs, several participants in the CHG group chose to withdraw from the study due to local pain and discomfort. As a result, recruitment was discontinued after enrolling 12 participants in the CHG group, and 11 in the FLZ group. Ultimately, only 5 participants in the CHG group completed the study as planned, while the remaining 7 discontinued participation at various stages (Fig 1).

### Acute treatment phase (Visit 1–2)

During the acute treatment phase, one participant in the CHG group withdrew from the study due to an AE (Fig 1). All participants in the FLZ group achieved microbiological cure, with negative *C. albicans* culture at Visit 2. In comparison, 9 out of 11 participants in the CHG group had negative cultures at Visit 2, indicating no statistically significant difference between the groups. Symptom scores, assessed via a standardized questionnaire, showed a significant reduction from baseline (Visit 1) to Visit 2 in both groups. In the FLZ group, the median symptom score decreased from 4 to 1 (p < 0.001), while in the CHG group, it decreased from 4 to 2 (p < 0.01). There was no statistically significant difference in symptom score between the two groups (Table 2). Similarly, examination scores, based on clinical signs observed during gynecological examination, also improved in both groups. In the FLZ group, the median score decreased from 2 to 1 (p = 0.05), and in the CHG group, from 2 to 0 (p < 0.001). Again, no significant difference was observed between the groups in terms of examination score (Table 2), but all findings need to be viewed cautiously because of the limited sample size.

**Table 2:** Results after acute and prophylactic treatment with either CHG or FLZ.

	Baseline		1 week (end of acute treatment)		3 months (end of prophylactic treatment)		6 months (follow-up visit)	
	CHG (n = 12)	FLZ (n = 11)	CHG (n = 11)	FLZ (n = 11)^‡^	CHG (n = 5)	FLZ (n = 10)^¶^	CHG (n = 5)	FLZ (n = 11)
Number or median (% or range)								
**Positive fungal culture**	12 (100)	11 (100)	2 (18)	0 (0)	2 (40)	2 (20)	3 (60)	4 (36)
Comparison^†^ between groups	ns		ns		ns		ns	
Compared to baseline	na		***	***	*	***	ns	**
**Symptom score (0**–**5)**	4 (2–5)	4 (2–5)	2 (0–4)	1 (0–4)	0 (0–3)	1 (0–4)	2 (0–5)	1 (0–4)
Comparison between groups	ns		ns		ns		ns	
Compared to baseline	na		**	***	**	***	ns	***
**Examination score (0**–**5)**	2 (1–4)	2 (0–5)	0 (0–5)	1 (0–4)	0 (0–4)	0 (0–3)	0 (0–3)	0 (0–4)
Comparison between groups	ns		ns		ns		ns	
Compared to baseline	na		***	ns	ns	**	ns	**
**Reinfection between visits** ^**¶¶**^	na		na		3 (60)	2 (20)	4 (80)	7 (64)
Comparison between groups					ns		ns	

**Results after treatment** Fungal cultures were performed according to clinical routine. Symptom score: vulvovaginal discharge = 1p, itching = 1p, dryness = 1p, burn = 1p, pain = 1p, self-reported. Clinical score: vulvovaginal redness = 1p, discharge = 1p, dryness = 1p, fissures = 1p, hyphae on wet smear = 1p, as assessed by the examining gynecologist. The number of reinfections between visits was culture-verified through self-sampling. ^†^Fischer’s exact test was applied to compare the number of positive fungal cultures and reinfections between study groups, and a Mann-Whitney U-test was applied for symptom score and examination score, *p* < 0.05 was considered statistically significant, significant *p*-values are marked with an asterisk; * p < 0.05, ** p < 0.01, *** p < 0.001. ^‡^One fungal culture from the FLZ group is missing from the 1-week visit. ^¶^One participant in the FLZ group missed the 3 months visit. ^¶¶^One participant in the FLZ group did not answer this question at the 3-months visit. Abbreviations: CHG = chlorhexidine gluconate, FLZ = fluconazole, na = not applicable, no. = number, ns = non-significant.

### Prophylactic treatment phase (Visit 2–3)

During the prophylactic treatment phase, an additional six participants in the CHG group discontinued the study due to AEs (Fig 1). At Visit 3, following three months of weekly prophylactic treatment, 8/10 participants in the FLZ group had negative *C. albicans* cultures, compared to 3/5 in the CHG group, indicating no statistically significant differences between the groups. However, it is important to consider that only 5 patients in the CHG group were left after the treatment phase. Symptom scores remained low in both groups, with a median score of 0 in the CHG group and 1 in the FLZ group. Both groups continued to show significant improvement in symptoms compared to baseline (Table 2). Examinations scores were also low at Visit 3, with a median 0 in both groups. Participants in the FLZ group demonstrated a statistically significant reduction in clinical signs of *Candida* infection from baseline to Visit 3 (p < 0.01), whereas this improvement was not statistically significant in the CHG group (Table 2).

Between Visit 2 and Visit 3, culture-verified recurrences were observed in 3/5 participants in the CHG group and in 2/10 participants in the FLZ group (Table 2). All participants with confirmed reinfections received a one-week course of the same treatment they had originally been allocated, provided it had been effective and well tolerated.

### Observation Phase (Visit 3–4)

During the three-month observation phase, several participants experienced new symptoms indicative of recurrence. Between Visit 3 and Visit 4, 4/5 participants in the CHG group and 7/11 in the FLZ group required additional treatment due to culture-verified reinfections. At Visit 4, *C. albicans* were detected in 3/5 of the CHG group and 4/11 of the FLZ group (ns) (Table 2). In the FLZ group, the proportion of participants with positive cultures at Visit 4 was significantly lower compared to baseline (p < 0.01), indicating sustained microbiological benefit. This reduction was not statistically significant in the CHG group. Symptom and examination scores at Visit 4 in the FLZ group remained comparable to those observed after the acute treatment phase, with significant reductions from baseline. In contrast, the CHG group showed an increase in symptom scores compared to Visit 3, and these were no longer significantly different from baseline. Additionally, no significant change in examination scores was observed in the CHG group at Visit 4 compared to baseline (Table 2). Again, the reduced sample size at the end of the study needs to be taken into consideration when interpreting the result.

### Vaginal Microbiome analyses

**Fig 3:**
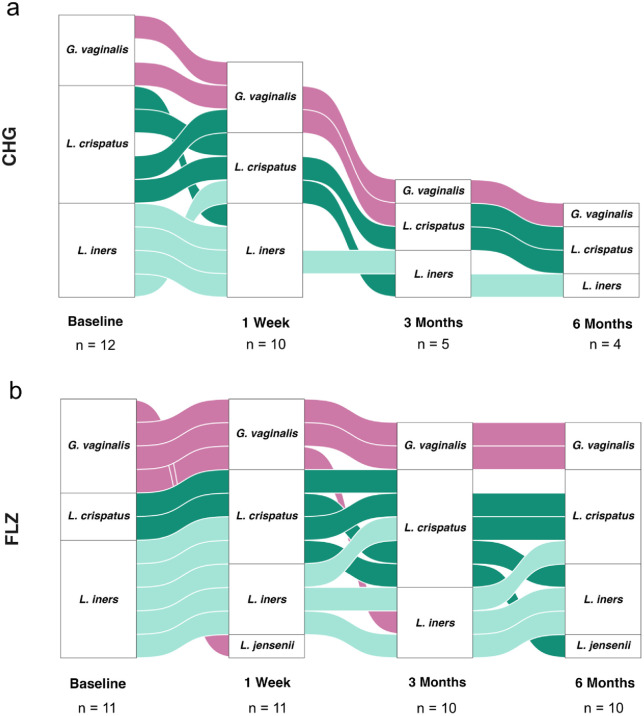
Major species shift between gynaecological examinations. An alluvial representation of the most abundant species per patient sample, across all gynaecological examination timepoints, for treatment with either **a** chlorhexidine or **b** fluconazole. CHG: chlorhexidine, FLZ: fluconazole.

In both treatment groups, the most dominant species identified in the vaginal microbiome were *Gardnerella vaginalis*, *Lactobacillus crispatus,* and *Lactobacillus iners*. The prevalence of *G. vaginalis* dominance decreased during treatment in both groups, although it persisted in a subset of participants despite either FLZ or CHG therapy (Fig 3).

In the CHG group, modest shifts in microbial dominance were observed throughout the study, without the emergence of new dominant species. At baseline, microbiome profiles showed dominance by *G. vaginalis* in 3 participants *L. crispatus* in 5, and *L. iners* in 4. After one week of treatment, two participants exhibited a shift in dominance between *L. crispatus* and *L. iners,* while *G. vaginalis* remained dominant, and even increased, in one participant. By the three-month timepoint, one participant transitioned from *G. vaginalis* to *L. crispatus* dominance, while another shifted from *L. crispatus* to *L. iners*. No further changes in dominant species were observed between the three- and six-month visits (Fig 3a).

In comparison, the FLZ treatment group showed more pronounced shifts toward a favorable vaginal microbiome composition. At baseline, 4 participants had microbiomes dominated by *G. vaginalis*, 2 by *L. crispatus,* and 5 by *L. iners*. Following treatment, two participants transitioned from *G. vaginalis* dominance to a *Lactobacill*us-dominated profile, one after one week and another by the three-month timepoint. Notably, *L. crispatus* became increasingly dominant over time. Between baseline and three months, 3 out of 5 participants with initial *L. iners* dominance shifted to *L. crispatus*. Additionally, *L. jensenii* emerged as the most abundant species in one participant after acute treatment and reappeared in another at the six-month follow-up (Fig 3b).

Overall, CHG treatment did not appear to harm the vaginal microbiome but had limited impact on promoting *L. crispatus* dominance. In contrast, FLZ treatment was more effective in inducing a shift toward a favorable *L. crispatus-*dominated microbiome.

## Discussion

The aim of this study was to conduct a non-inferiority RCT to evaluate the efficacy of vaginal CHG as an alternative to the standard oral FLZ treatment for women with RVVC. The rationale was grounded in previous explorative studies suggesting the potential of CHG as a non-azole treatment option, with the goal of minimizing systemic side effects associated with long-term FLZ use [11,12].

However, the study was prematurely terminated due to a high frequency of local AEs reported by participants in the CHG group, primarily vaginal burning and discomfort. As a result, the final sample size did not meet the requirements established in the original power calculation. Consequently, the study transitioned from a full-scale RCT to a pilot study, and the findings should be interpreted with caution. Despite these limitations, several important observations emerged.

In the FLZ group, all participants cleared the infection after one week of acute treatment, compared to 9/11 in the CHG group. During the prophylactic and observational phases, recurrences were observed in both groups, but were more frequent among CHG participants, particularly toward the end of the study. Symptoms of RVVC improved in both groups over the course of the study. However, at the final visit, participants in the FLZ group continued to report fewer symptoms compared to baseline, a trend not observed in the CHG group. Although the differences between groups did not reach statistical significance in favor of FLZ, it is important to consider that the outcomes might have differed if the full cohort of planned participants had been included.

The results also support the characteristic features of RVVC, notably its frequent recurrences with often unidentified underlying causes [3]. Several hypotheses have been proposed to explain RVVC, including alterations in the vaginal immune response that increase host susceptibility, specific virulence traits of the Candida strains involved, and genetic polymorphisms [5,7]. One key fungal virulence factor is biofilm formation, which is associated with increased resistance to antifungal treatments [30]. Previous studies suggest that CHG may exert its antifungal effects both through direct fungicidal activity and by disrupting vaginal biofilms [15]. However, the current CHG vaginal formulation is not suitable for clinical use due to local irritation. Despite this limitation, we believe that alternative pharmaceutical preparations of CHG warrant further investigation.

Concerns have been raised for increasing resistance to FLZ [31,32], although current global data do not indicate a clear trend of rising prevalence. In a retrospective study conducted in the United States, 7.3% of 970 *Candida* isolates exhibited resistance to FLZ [33]. Similarly, in Sweden, FLZ resistance is increasingly reported among patients with RVVC caused by *C. albicans*, with a prevalence of 10.5% according to E. Chryssantou (personal communication, Department of Microbiology Karolinska University Hospital 2024). In our study, 16% of the tested *C.* *albicans* strains demonstrated reduced susceptibility or resistance to FLZ. This finding underscores the urgent need to explore and develop alternative therapeutic options for RVVC, particularly in light of the potential for antifungal resistance to compromise current treatment efficacy. Development of other local applied anti-fungal agents could represent a promising avenue for future treatment strategies targeting recurrent infections. Intravaginal suppositories of boric acid are used as an alternative treatment, but the exact mode of action is not known. Unlike FLZ, boric acid has been shown to inhibit hyphal formation and reduced established biofilm [34]. In our previous *in vitro* study, CHG showed a similar action on preventing biofilm formation along with fungicidal activity [15]. In that perspective, CHG could be another non-azole option for RVVC in the future if a tolerable formula of the drug is established.

There are relatively few studies examining the impact of CHG on the vaginal microbiome. One human study reported a reduction in *Lactobacillus* species following nightly vaginal application of 0.5% CHG for one week. However, the vaginal flora had largely recovered within 30 days, and AEs were minimal [35]. In an animal study, two vaginal applications of 0.25% CHG administered 24 hours apart did not result in significant alterations of the vaginal microbiota [36]. In our study, no harmful changes to the vaginal microbiome were observed in the CHG group. However, it also did not promote a notable shift toward *L. crispatus* dominance, a microbial profile generally associated with vaginal health. In contrast, FLZ treatment more effectively promoted a shift toward *L*. *crispatus* dominance. This suggests that while CHG may be microbiologically safe, however, larger studies are needed to investigate its influence on restoring or promoting a favorable vaginal microbiome may be limited compared to FLZ.

The primary limitation of this study was the unexpected occurrence of local irritation associated with CHG treatment, which necessitated early termination of the trial before reaching the intended sample size. As a result, we cannot conclusively determine whether CHG is non-inferior to FLZ in terms of efficacy for clearing acute C. *albicans* infections or preventing recurrences. Nevertheless, CHG did not appear to negatively impact the vaginal microbiome, and the treatment outcomes observed in this study suggest a potential comparable efficacy to FLZ. Given these findings, we propose that further research should explore the development of alternative, less irritative vaginal formulations of CHG. Such formulations could allow for a more comprehensive evaluation of CHG’s antifungal and biofilm-disrupting properties. This approach may offer a promising alternative for the treatment of RVVC, particularly in the context of rising concerns about FLZ resistance.

## Supporting information

S1 Table One participant in the FLZ group missed the 3 months visit. Abbreviations: CHG = chlorhexidine gluconate, FLZ = fluconazole. Adverse events reported after acute and prophylactic treatment with either CHG or FLZ.
(DOCX)

S2 Table Result per participant. ^¶^
(XLSX)

S1 File Study protocol. (DOCX)

S2 File Checklist noninferiority trials. (DOCX)
