# Supplementary material for: Vaginal chlorhexidine gluconate versus fluconazole for recurrent vulvovaginal candidiasis: A randomized noninferiority trial
Source: PLoS One. 2026 Jan 20;21(1):e0340862. doi: 10.1371/journal.pone.0340862 (PMC12818662; doi:10.1371/journal.pone.0340862)
Supplement: S1 Table — Adverse events reported after acute and prophylactic treatment with either CHG or FLZ. (DOCX) [file pone.0340862.s001.docx]

**Supplementary Table S1. Adverse events reported after acute and prophylactic treatment with either CHG or FLZ**

|  | **1 week**  **(end of acute treatment)** | | **3 months**  **(end of prophylactic treatment)** | |
| --- | --- | --- | --- | --- |
|  | **CHG**  **(n=11)** | **FLZ (n=11)** | **CHG (n=5)** | **FLZ** **(n=10) ^¶^** |
|  | Number (%) | | | |
| **Local side effects** |  |  |  |  |
| Vulvovaginal itching | 6 (55) | 0 (0) | 1 (20) | 0 (0) |
| Vulvovaginal burn | 7 (64) | 0 (0) | 0 (0) | 0 (0) |
| Vulvovaginal pain | 3 (27) | 0 (0) | 0 (0) | 0 (0) |
| Vulvovaginal discharge | 1 (9) | 0 (0) | 0 (0) | 0 (0) |
| **Systemic side effects** |  |  |  |  |
| Headache | 0 (0) | 1 (9) | 0 (0) | 1 (10) |
| Nausea | 0 (0) | 2 (18) | 0 (0) | 0 (0) |
| Diarrhea | 0 (0) | 1 (9) | 0 (0) | 0 (0) |

^¶^One participant in the FLZ group missed the 3 months visit. Abbreviations: CHG = chlorhexidine gluconate, FLZ = fluconazole.
