# Supplementary material for: Vaginal chlorhexidine gluconate versus fluconazole for recurrent vulvovaginal candidiasis: A randomized noninferiority trial
Source: PLoS One. 2026 Jan 20;21(1):e0340862. doi: 10.1371/journal.pone.0340862 (PMC12818662; doi:10.1371/journal.pone.0340862)
Supplement: S1 File — (DOCX) [file pone.0340862.s003.docx]

CLINICAL STUDY PROTOCOL

| **A RCT for chlorhexidine gluconate as treatment and prophylaxis for Recurrent Vulvovaginal Candidiasis**  Chlorhex | |
| --- | --- |
|  |  |
| Study code: | Chlorhex-KKDS-2021 |
| EudraCT number: | 2020-000758-81 |
| Version number: | Version 2 |
| Date: | 2021-02-05 |
|  |  |
| Sponsor: | Karolinska Institutet |
|  | |
| Principal Investigator | Nina Bohm-Starke,  Dept of Obstetrics & Gynecology  Danderyd Hospital  SE-182 88 Stockholm, Sweden  nina.bohm-starke@sll.se |
|  |  |
|  |  |

Table Of Contents

[Signature page 5](#_Toc62024028)

[Contact information 6](#_Toc62024029)

[List of used acronyms and abbreviations 7](#_Toc62024030)

[1. Synopsis 9](#_Toc62024031)

[2. Background and rationale 12](#_Toc62024032)

[3. Benefit-risk evaluation 13](#_Toc62024033)

[4. Study objectives 14](#_Toc62024034)

[4.1. Primary objective 14](#_Toc62024035)

[4.2. Secondary objective(s) 14](#_Toc62024036)

[4.3. Primary endpoint (variable) 14](#_Toc62024037)

[4.4. Secondary endpoint (variables) 15](#_Toc62024038)

[5. Study design and procedures 15](#_Toc62024039)

[5.1. Overall study design 15](#_Toc62024040)

[5.2. Procedures and flow chart 17](#_Toc62024041)

[5.3. Biological sampling procedures 20](#_Toc62024042)

[5.3.1. Handling, storage, and destruction of biological samples 20](#_Toc62024043)

[5.3.2. Total volume of blood per subject 20](#_Toc62024044)

[5.3.3. Biobank 20](#_Toc62024045)

[5.4. End of Study 20](#_Toc62024046)

[6. Subject selection 20](#_Toc62024047)

[6.1. Inclusion criteria 20](#_Toc62024048)

[6.2. Exclusion criteria 21](#_Toc62024049)

[6.3. Screening 21](#_Toc62024050)

[6.4. Withdrawal criteria 21](#_Toc62024051)

[7. Study treatments 21](#_Toc62024052)

[7.1. Description of investigational product(s) 21](#_Toc62024053)

[7.2. Dose and administration 22](#_Toc62024054)

[7.3. Packaging, labeling, and handling of investigational products(s) 22](#_Toc62024055)

[7.4. Drug accountability and treatment compliance 23](#_Toc62024056)

[7.5. Randomization 23](#_Toc62024057)

[7.6. Blinding 23](#_Toc62024058)

[7.7. Code breaking 24](#_Toc62024059)

[7.8. Concomitant medications 24](#_Toc62024060)

[7.9. Destruction 24](#_Toc62024061)

[7.10. Treatment after study end 24](#_Toc62024062)

[8. Methods for measurement of endpoints for clinical efficacy and safety 24](#_Toc62024063)

[8.1. Methods for measurement of endpoints for clinical efficacy 24](#_Toc62024064)

[8.1.1. Primary endpoint (variable) 24](#_Toc62024065)

[8.1.2. Secondary endpoints (variables) 24](#_Toc62024066)

[8.2. Methods for measurement of endpoints (variables) for clinical safety 26](#_Toc62024067)

[9. Handling of Adverse Events 26](#_Toc62024068)

[9.1. Definitions 26](#_Toc62024069)

[9.1.1. Adverse Event (AE) 26](#_Toc62024070)

[9.1.2. Adverse Reaction (AR) 26](#_Toc62024071)

[9.1.3. Serious Adverse Event (SAE) 26](#_Toc62024072)

[9.1.4. Suspected Unexpected Serious Adverse Reaction (SUSAR) 27](#_Toc62024073)

[9.2. Assessment of Adverse Events 27](#_Toc62024074)

[9.2.1. Assessment of causal relationship 27](#_Toc62024075)

[9.2.2. Assessment of intensity 27](#_Toc62024076)

[9.2.3. Assessment of seriousness 28](#_Toc62024077)

[9.3. Reporting and registration of Adverse Events 28](#_Toc62024078)

[9.3.1. Reporting of Serious Adverse Events (SAE) 28](#_Toc62024079)

[9.3.2. Reporting of Suspected Unexpected Serious Adverse Reactions (SUSAR) 28](#_Toc62024080)

[9.4. Follow-up of Adverse Events 29](#_Toc62024081)

[9.5. Annual Safety Report (Development Safety Update Report, DSUR) 29](#_Toc62024082)

[9.6. Procedures in case of emergencies, overdose or pregnancy 29](#_Toc62024083)

[9.7. Reference Safety Information 29](#_Toc62024084)

[10. Statistics 29](#_Toc62024085)

[10.1. Analysis population 29](#_Toc62024086)

[10.2. Statistical analyses 29](#_Toc62024087)

[10.2.1. Statistical methods 29](#_Toc62024088)

[10.2.2. Drop-outs 31](#_Toc62024089)

[10.3. Adjustment of significance and confidence interval 31](#_Toc62024090)

[10.4. Sample size calculations 31](#_Toc62024091)

[10.5. Interim analysis (if relevant) 31](#_Toc62024092)

[11. Quality Control and Quality Assurance 31](#_Toc62024093)

[11.1. Quality Assurance and Sponsor oversight 31](#_Toc62024094)

[11.2. Monitoring 32](#_Toc62024095)

[11.3. Source data 32](#_Toc62024096)

[11.4. Deviations or serious breaches 33](#_Toc62024097)

[11.5. Audits and inspections 33](#_Toc62024098)

[12. Ethics 33](#_Toc62024099)

[12.1. Compliance to the protocol, GCP and regulations 33](#_Toc62024100)

[12.2. Ethical review of the study 33](#_Toc62024101)

[12.3. Procedure for obtaining informed consent 34](#_Toc62024102)

[12.4. Data protection 34](#_Toc62024103)

[12.5. Insurances 34](#_Toc62024104)

[13. Substantial changes to the study 34](#_Toc62024105)

[14. Collection, handling, and archiving data 35](#_Toc62024106)

[14.1. Case Report Form (Forskningspersonsformulär) 35](#_Toc62024107)

[Each study visit will also be registered in the hospital’s medical records 36](#_Toc62024108)

[15. Notification of study completion, reporting, and publication 36](#_Toc62024109)

[16. References 36](#_Toc62024110)

[17. Attachments 37](#_Toc62024111)

# Signature page

**Sponsor/Principal Investigator**

I am responsible for ensuring that this protocol includes all essential information to be able to conduct this study. I will submit the protocol and all other important study-related information to the responsible investigator(s) so that they can conduct the study correctly. I am aware that it is my responsibility to hold the staff members who work with this study informed and trained.

| Sponsor’s signature Date  Nina Bohm-Starke |
| --- |

Printed name

# Contact information

| **Role** |  |
| --- | --- |
| Sponsor | Karolinska Institutet  Danderyd Hospital  182 88 Stockholm. Sweden  +46 8 12356695 |
| Principal Investigator | Nina Bohm-Starke  Department of Obstetrics & Gynecology Danderyd Hospital  182 88 Stockholm, Sweden  nina.bohm-starke@sll.se |
| Investigator | Cathrin Alvendal  Department of Obstetrics & Gynecology Danderyd Hospital  182 88 Stockholm, Sweden  cathrin.alvendal@sll.se |
| Clinical monitoring organization | Department of Obstetrics & Gynecology  Danderyd Hospital  182 88 Stockholm, Sweden  Annelie Wikström, research midwife  annelie.wikstrom@sll.se |
| Study coordinator | Department of Obstetrics & Gynecology  Danderyd Hospital  182 88 Stockholm, Sweden  Helene Fagraeus, research midwife  helene.fagraeus@sll.se |

# List of used acronyms and abbreviations

| **Abbreviation** | **Term/Explanation** |
| --- | --- |
| AE | Adverse Event = any untoward medical occurrence |
| AR | Adverse Reaction = adverse event, that is each unfavorable and unexpected reaction to a study treatment, regardless of dose |
| CA | Competent Authorities |
| CI | Confidence interval |
| CIDAMP | Cationic intrinsically disordered antimicrobial peptides |
| CHG | Chlorhexidine gluconat |
| CRF | Case Report Form |
| DSUR | Development Safety Update Report = annual safety report |
| eCRF | Electronic Case Report Form |
| EPM | Etikprövningsmyndigheten (English: Swedish Ethical Review Authority) |
| FLZ | Fluconazole |
| GCP | Good Clinical Practice |
| GDPR | General Data Protection Regulation |
| ICH | International Council for Harmonization |
| IHC | Immunohistochemistry |
| ITT | Intention-to-treat = including all data from all subjects who have participated in the study |
| LVFS | Läkemedelsverkets författningssamling (English: Swedish Medical Products Agency’s statutes) |
| NSAID | Non Steroid Anti-Inflammatory Drug |
| PCR | Polymerase Chain Reaction |
| PP | Per Protocol analysis = including only data from subjects who have completed the study completely in accordance with the protocol, with no deviations from the protocol |
| RCT | Randomized clinical trial |
| RNA | Ribonucleic Acid |
| RVVC | Recurrent vulvovaginal candidiasis |
| SAE | Serious Adverse Event = serious untoward medical occurrence |
| SPC or SmPC | Summary of Product Characteristics |
| SUSAR | Suspected Unexpected Serious Adverse Reaction |

#

# Synopsis

The overall aim of this study is to investigate if vaginally applied 1% chlorhexidine gluconate (CHG) could be an alternative treatment to oral fluconazole (FLZ), both during an acute episode and as prophylaxis, against recurrent infections of vulvovaginal candidiasis (RVVC). If CHG is at least as effective as FLZ with little impact on vaginal lactobacillus, with high tolerability and without cytotoxic effect on epithelial cells, the results of the study might lead to major benefits to the patients with reduced risk of systemic side effects such as drug interactions, development of drug resistance and reduced drug costs.

| EudraCT number:  2020-000758-81 |  |
| --- | --- |
| Title: A RCT for chlorhexidine gluconate as treatment and prophylaxis for Recurrent Vulvovaginal Candidiasis |  |
| Study code: Chlorhex-KKDS-2021 |  |
| Short background/ Rationale/Aim:  Recurrent vulvovaginal candidiasis is common in young fertile women. There are limited treatment options and the recommended treatment of long-term oral fluconazole is not suitable for all patients. The overall aim of this study is to investigate if vaginally applied 1% chlorhexidine gluconate (CHG) is at least as effective as oral fluconazole, both during an acute episode and as prophylactic treatment for recurrent vulvovaginal candidiasis (RVVC). |  |
| Study objectives:  RCT to compare the treatment effect and  safety aspects of vaginal 1%chlorhexidine  gluconate (Hibitane® vaginal cream)  versus oral fluconazole (Fluconazole®)  for recurrent vulvovaginal candidiasis  (RVVC). | Primary objective:  To analyze if vaginal application of CHG is at least as effective as oral FLZ as treatment and prophylaxis of RVVC in terms of clearing the infection (negative culture for *Candida albicans*)  Secondary objectives:  To analyze adverse events  To analyzed clinical symptoms and findings  To study the event of relapses during and after the prophylaxis treatment  To study the effect on vaginal microbiome of lactobacillus before and after treatment and prophylaxis with CHG for RVVC.  To investigate if CHG have toxic effect on the vaginal epithelial cells.  To analyze vaginal biofilm formation before and after treatment and prophylaxis with CHG for RVVC. |
| Study design:  The study is a randomized open Phase II trial and the protocol is designed as a non-inferiority trial. |  |
| Study population:  Women with culture verified recurrent vulvovaginal *Candida albicans* infection |  |
| Number of subjects: 60 |  |
| Inclusion criteria:   - 18-50 years of age - A history of > 2 candida infections the last year - Symptoms of acute vulvovaginal candida infection - Culture verified infection with *Candida albicans* - Adequate contraceptive method - Able to understand oral and written information in Swedish - The subject has given written consent to participate in the study |  |
| Exclusion criteria:   - Severe somatic or mental illness (including liver and renal failure and cardiac disease) - Immunosuppressive medication - Pregnancy - Lactation - Other ongoing gynecological infections - Allergy to fluconazole or Chlorhexidine gluconate - Medication that might have impact on the QT interval (terfenadin, cisaprid, astemizol pimozid, kinidin, erythromycin, halofantrin, amiodaron) - Participation or recent participation (30 days) in a clinical study with an investigational product. Previous participation in this study. | |
| Investigational product(s), dosage, administration:  Chlorhexidine gluconate, 1% vaginal crème (Hibitane®), 8 ml, vaginal application  Fluconazole, oral capsules, 150 mg (control treatment) |  |
| **Study endpoints**:   1. To analyze if vaginal application of CHG is at least as effective as oral FLZ as treatment and prophylaxis of RVVC. 2. To analyze adverse events (AE) 3. To study the effect on vaginal microbiome of lactobacillus before and after treatment and prophylaxis with CHG for RVVC. 4. To study the event of relapses during and after the prophylaxis treatment 5. To investigate if CHG have toxic effect on the vaginal epithelial cells. 6. To analyze vaginal biofilm formation before and after treatment and prophylaxis with CHG for RVVC. | **Primary endpoint**:  The proportion of women in each group  that has cleared the infection after 1week  after treatment, defined as negative  cultures for C. *albicans.*  **Secondary endpoints**:   1. Negative cultures for *Candida albicans* at 3 months’ and 6 months’ follow-ups, after end of prophylactic treatment. 2. Analysis of proportion of women with adverse events (AE). 3. Proportion of women with symptom score > 2 (composite index). 4. Proportion of women with examination score >2 (composite index). 5. Proportion of women with reduced vaginal lactobacilli content in vaginal smears 6. Proportion of women with relapse of *Candida albicans* infection between end of treatment and end of prophylactic treatment (1week post treatment to 3 months’ follow-up) and between 3- and 6 months’ follow-up after end of prophylactic treatment 7. Proportion of women with possible toxic effect on the vaginal epithelium 8. Analysis of biofilm formation during and after treatment and prophylaxis. |
| Study period: | Q2 2021 – Q4 2024 |

# Background and rationale

Vulvovaginal fungal infections are very common, affecting approximately 75% of all women at some point in their lives [1]. Many women relapse and about 5-8% of fertile women suffer from repeated infections, or "recurrent vulvovaginal candida infection" (RVVC), defined as 3-4 infections per year [2, 3]. There are several known risk factors for sporadic fungal infections where antibiotic therapy, pregnancy and immunosuppression are the most common causes. Identified causes of RVVC are on the other hand unclear and the patients are in most cases otherwise healthy. This has prompted theories that the etiology to RVVC could be alterations of the vaginal immune system or specific virulent characteristics of the involved Candida strains [3]. Suffering from RVVC has a major impact on the quality of life of the affected women. For long periods, they cannot have pain free sex and they often suffer from constant irritation and uncomfortableness, which in turn can lead to anxiety, depression and possible development of provoked vestibulodynia [3, 4].

Up to six months of treatment with fluconazole (FLZ) is the recommended treatment for RVVC [5, 6]. Over the last ten years, the use of FLZ has increased markedly in many countries. So far, no major problems have been noted with resistance development, but there is concern that this will occur in the future and alternative treatments are requested [7]. This is one of the reasons why the Swedish Medical Products Agency reintroduced prescription requirements for single dose of FLZ in 2017. Although many patients tolerate the treatment well, there are reported side effects. In recent years, it has emerged that flukonazol interacts with several different types of drugs that are common in the patient group; several antidepressants, pain relief at dysmenorrhea (NSAID) and oral contraceptives to name a few [8]. The most serious interaction is the risk of extended QT range and cardiac arrhythmia. In addition, FLZ is not approved during pregnancy, which in itself poses an increased risk of vulvovaginal fungal infections. [8].

Our research group has previously done an *in vitro* study in which we analyzed the effect of FLZ and the antiseptic substance chlorhexidine gluconate’s (CHG) ability to kill fungal cells and to break down existing biofilm or prevent new biofilm formation [9]. The biofilm formation is an important stage for the fungal cells to attach to surfaces such as skin and mucosa and is considered a first step in the development of an infection [10]. In the biofilm, the fungus can hide from the immune system and also to some extent for various treatments aimed against the fungus. The results of the study showed that CHG was better than FLZ both at killing the fungal cells and preventing new biofilm from forming and dissolving already established "old" biofilm. This effect is absolutely crucial for successful treatment with antimycotics. These encouraging results form the basis of the planned study.

Hibitane® is an over-the-counter vaginal cream consisting of 1% chlorhexidine gluconate with the indication antiseptic use in vaginal examinations, especially during childbirth. The product has been used for a long time in various gynecological and obstetric surgical procedures. Hibitane® is approved during pregnancy and the cream is usually well tolerated [11]. There are only few studies that investigated how vaginal normal flora is affected by CHG. One human study showed a decrease in the number of lactobacilli a week after the vaginal application of 0.5% CHG every night for a week. After 30 days, the vaginal normal flora was basically fully recovered and the side effects were few [12]. In an animal study, no major effect was seen on the vaginal flora after 2 vaginal applications of CGH 0.25% at 24 hours apart [13].

Many women with RVVC are referred to the vulvar clinic at the gynecological department at Danderyd Hospital in Stockholm. We have followed these women clinically for many years and have also performed several scientific studies. Our clinical experience is fully in line with the described high risk of infection relapse and the need for repeated and long use of fluconazole. Several of our patients have problems with side effects of the medication and there are also difficulties in combining fluconazole with other concomittant medications commonly used by this group of patients. If Hibitane® is at least as effective as fluconazole with little impact on vaginal lactobacillus, with high tolerablility and without cytotoxic effect on epithelial cells, the results of the study might lead to that an already established and inexpensive drug can be used by a large group of young women with a new indication.

# Benefit-risk evaluation

In the study we will examine an already approved drug with basically the same indication described in FASS, i.e. antiseptic effect. According to the product recap available for Hibitane® vaginal cream, the only contraindication is contact of active substance with brain or meningeal tissue, or ear canal due to neurotoxicity. Hibitane® is approved for vaginal use even during pregnancy and lactation and is usually tolerated well in the vagina but can provide some skin and mucosal irritation. Allergic reactions are rare. Thereby the possible risks for the patients is considered to be low. The reference treatment is oral fluconazole, which will be used in doses and duration according with administration specified in FASS. There are known AE that could occur during treatment with fluconzole, but the most serious ones are eliminated by the exclusion criteria of the study.

Since vaginal Hibitane® treatment has not been evaluated for vulvovaginal candida infections *in vivo*, there is a risk that the patients will not be optimally treated against their candida infection if Hibitane® turns out to have limited treatment efficacy. However, all patients in the study will have the opportunity for additional follow-ups and optional treatment after the study if they should not recover from the study treatment they have been allocated to.

The gynecological examinations, including vaginal sampling, are conducted according to customary routines. Small (3-4 mm) vaginal biopsies will be taken during the study and may cause some pain associated with the anesthetic, but the biopsy and healing itself should not pose any major risks of bleeding or infection. The research team has previously taken similar biopsies without any complications.

The benefit of the study will mainly be on group level rather than individual benefit. The aim of the study is to be able to offer patients with RVVC an alternative treatment to oral fluconazole. The major long-term benefits for the patients would therefore be reduced risk of systemic side effects and interactions with other medications as well as hampering drug resistance.

Further ethical consideration is handling of patient data. Before entering the study, all patients will sign an orally and written informed consent and receive a study specific code. All the results will be reported on group level and no individual patient will be identified, for more details, please see section 14.

# Study objectives

The study is a RCT to compare the treatment effect and safety aspects of vaginal 1% Chlorhexidine gluconate (CHG) versus oral Fluconazole® (FLZ) for recurrent vulvovaginal candidiasis (RVVC).

## Primary objective

To analyze if daily vaginal application of 8 ml of Hibitane® for one week and thereafter once a week for another 11 consecutive weeks is at least as effective as fluconazole 150 mg capsules every third day for the first three doses and thereafter 150 mg once a week for another 11 consecutive weeks regarding treatment- and prophylactic efficacy for recurrent vulvovaginal *Candida albicans* infection*.*

## Secondary objective(s)

1. To analyze adverse events
2. To analyzed clinical symptoms and findings
3. To study the effect on vaginal microbiome of lactobacillus before and after treatment and prophylaxis with CHG for RVVC.
4. To evaluate the event of relapses during and after the prophylaxis treatment
5. To investigate if CHG has toxic effect on the vaginal epithelial cells.
6. To analyze vaginal biofilm formation before and after treatment and prophylaxis with CHG and FLZ for RVVC.

## Primary endpoint (variable)

The proportion of women in each group that has negative vaginal cultures for *Candida albicans* 1 week after active treatment.

## Secondary endpoint (variables)

1. The proportion of women in each group that has negative vaginal cultures for *Candida albicans* at 3 months’ follow-up after prophylactic treatment and at 6 months’ follow-up after the observational phase of the study.
2. Analysis of proportion of women with adverse events (AE) across treatment arms. The AEs will also be reported in descriptive terms and summarized for each treatment arm.
3. Proportion of women with symptom score > 2. Each symptom of typical discharge, itching, dryness of the skin/mucosa, burning and pain will generate 1 point of a composite index (range 0-5).
4. Proportion of women with examination score >2. Each finding of redness skin/mucosa, typical discharge, dry skin/mucosa, fissures skin/mucosa, visible candida hyphae in the microscope will generate 1 point of a composite index (range 0-5).
5. Proportion of women with reduced vaginal lactobacilli content in vaginal smears measured by a semi-quantitative method as normal or reduced quantity.
6. Proportion of women with relapse of *Candida albicans* infection between end of treatment and end of prophylactic treatment (1week post treatment to 3 months’ follow-up) and between 3- and 6 months’ follow-up after end of prophylactic treatment.
7. Proportion of women with possible toxic effect on the vaginal epithelium, defined as disruption and/or inflammation of the epithelial lining, analyzed from vaginal biopsies before and after treatment and prophylaxis.
8. Further analysis of mucosa inflammatory mechanism (histopathology and immunohistochemistry) and biofilm formation during and after treatment and prophylaxis, analyzed from vaginal biopsies. The methods for these *in vitro* exploratory analyses are not yet decided on.

# Study design and procedures

The study was originally planned to start in March 2021, but the start of the study has been postponed due to the Covid -19 pandemic. The study will not start until the pandemic is under control and when there are no risks for participants to travel and visit Danderyd hospital. Estimated study start will be May 2021, when the Covid -19 vaccination is carried out in the major part of the Stockholm population. Caution to avoid infection transmission will be carried out and recommendations and regulations from the Swedish Health Authority and from Region Stockholm will be followed.

## Overall study design

The study is a 6 months clinical Phase II trial to compare if 1% chlorhexidine gluconate vaginal cream (Hibitane®) is at least as effective and safe as fluconazole (reference treatment) for culture-verified recurrent vulvovaginal candidiasis. The study is a randomized open non-inferiority trial with parallel treatment groups. The trial is not possible to blind for neither participants nor investigators due to the differences between study treatments. There is low risk of bias since the primary outcome variable is objective (negative vaginal culture for *Candida albicans*).

The rational for the study is to find an alternative treatment to fluconazole for RVVC with high tolerability and without cytotoxic effect on epithelial cells and negative impact on the vaginal microbes.

The participants will be randomization to;

- Investigational medication with Hibitane® vaginal cream 8 ml every night for a week and then prophylactic treatment with 8 ml/week for another 11 weeks

or

- Reference treatment with Fluconazole® 150 mg (oral capsule) every 3 days for the first 3 doses, then prophylactic treatment with 150 mg/week for another 11 weeks

Please see section 7 for more details on randomization and study treatments.

Figure 1 Study design

| **Visit** | **1** | **2** | **3** | **4** | **5** |
| --- | --- | --- | --- | --- | --- |
|  |  | CHG | Chlorhexidine gluconate  vaginal application  8 ml/w, 11 w | |  |
|  |  | 8 ml/day,1 w  R |  |  | N=30 |
|  |  |  | Fluconazole  oral capsules  150 mg/w, 11 w | |  |
|  |  | FLZ  150 mg/day,1w |  |  | N=30 |
| **Week:** | **-1** | **0** | **1** | **12** | **24** |
|  | Screening | Inclusion/  Baseline  Randomization | Follow-up | Follow-up | Follow-up and end of treatment |

##

## Procedures and flow chart

**Five research visits are planned:**

**Visit 1** Screening – oral and written study information, signing of informed consent, vaginal culture for Candida albicans and chlamydia/gonorrhea (PCR test). 45 min.

**Visit 2** Inclusion (baseline), within 1 week after Visit 1. Control for inclusion/exclusion criteria, randomization to study medication after positive culture for *Candida albicans* and negative chlamydia/gonorrhea (PCR test) – health survey, control of concomitant medications, examination, vaginal biopsies, pregnancy test and control of adequate contraceptive methods to avoid pregnancy throughout the study. 1 h.

**Visit 3** 1 week (+2 days) after completed treatment. Control of AE, culture for *Candida albicans* and examination (no biopsies). 30 min.

**Visit 4** After 12 weeks (+ 1 week) from inclusion when prophylactic treatment is completed. Control of AE and relapses, culture for *Candida albicans* and examination, vaginal biopsies. 45 min.

**Visit 5** Follow-up 6 months (+ 1-2 weeks) from baseline/inclusion. Control of AE and relapses, culture for *Candida albicans* and examination (no biopsies). End of study. 30 min.

A weekly web-based dairy (eCRF (Entermedic)) will be used for follow-up of treatment

compliance, efficacy and adverse events. A personal link will be sent via e-mail every week

from week 2-24. During the prophylactic treatment (Visits 2-3) and during the observational

phase of the study (Visit 4-5), the participants are asked to report any suspicious relapses in

the dairy and contact the research midwife if needed. They will also receive equipment for

vaginal cultures that can be used for self-sampling at home to detect true relapses. In case of

positive cultures for *Candida albicans*, they will be offered the same medication as previously

used in the study if the treatment was effective and no adverse events occurred. Otherwise,

an individual treatment option will be used.

Table 1 Flow chart

| **Procedure** | **Visit 1**  Screening  Week -1 | **Visit 2**  Baseline/  inclusion  Week 0  (within 1 w from Visit 1) | **Visit 3**  Follow-up  Week 1  (treatment)  (+2 d) | **Visit 4**  Follow-up  Week 12  (prophylactic phase)  (+1 w) | **Visit 5**  Follow-up  Week 24  (observational phase)  (+2 w) |
| --- | --- | --- | --- | --- | --- |
| Incl/exclusion criteria | √ | √* |  |  |  |
| Informed signed consent | √ |  |  |  |  |
| Medical health questionnaire/ concomitant medications |  | √ |  |  |  |
| Randomization |  | √ |  |  |  |
| Vaginal culture  Candida | √ |  | √ | √ | √ |
| PCR test**  Clamydia/  Gonorrhea | √ |  |  |  |  |
| Pregnancy test |  | √ | √ |  |  |
| Symptom score |  | √ | √ | √ | √ |
| Examination score |  | √ | √ | √ | √ |
| Microscopy  lactobacilli |  | √ | √ | √ | √ |
| Vaginal biopsies |  | √ |  | √ |  |
| Instructions for handling the investigational product(s) |  | √ |  |  |  |
| Adverse Events (AE & SAE) |  |  | √ | √ | √ |
| Study end |  |  |  |  | √ |

**if positive culture for Candida albicans*** Chlamydia/gonorrhea test will be repeated in case of new partner during the study

##

## Biological sampling procedures

### Handling, storage, and destruction of biological samples

Three vaginal forceps biopsies of 3-4 mm will be taken at Visit 2 and Visit 4 after local anesthesia. One biopsy will be fixed in formalin and used for pathophysiological examination and immunohistochemical (IHC) analysis. A second biopsy will be placed in RNA*later* RNA Stabilization Reagent (Qiagen) and subjected to RNA isolation and gene expression analysis. The third biopsy will be used to study biofilm formation. The laboratory work will be performed at the core facilities at Karolinska Institutet.

### Total volume of blood per subject

N/A

### Biobank

All biopsy samples taken in this study will be registered in “Stockholm medical biobank” (register number 914, sample ID Bb142563) and handled according to the current biobank laws and regulations. The samples are coded/pseudonymized to protect the subject´s identification. All samples and the identification/code list are stored securely and separately to prevent unauthorized persons from having access to them.

## End of Study

The study ends when the last subject has completed the last follow-up. The study may be prematurely terminated if it appears that the treatment involved a large number of serious adverse events (SAE) or if recruitment of subjects cannot be met within reasonable time limits. If the study is prematurely terminated or suspended, the investigator should immediately inform the subjects about this and ensure appropriate treatment and follow-up. The regulatory authority should be informed as soon as possible, but no later than within 15 days.

Decisions on premature termination are taken by the sponsor.

# Subject selection

## Inclusion criteria

To be included in the study, subjects must meet the following criteria:

- 18-50 years of age
- A history of > 2 candida infections the last year
- Symptoms of acute vulvovaginal candida infection
- Culture verified infection with *Candida albicans*
- Adequate contraceptive method
- Able to understand oral and written information in Swedish
- The subject has given written consent to participate in the study

## Exclusion criteria

Subjects must not be included in the study if any of the following criteria are met:

- Severe somatic or mental illness (including liver and kidney failure and cardiac disease)
- Immunosuppressive medication
- Pregnancy
- Lactation
- Other ongoing gynecological infections
- Allergy to fluconazole or chlorhexidine gluconate
- Citalopram or other medication that might have impact on the QT interval (terfenadin, cisaprid, astemizol, pimozid, kinidin, erythromycin, halofantrin, amiodaron)
- Participation or recent participation (30 days) in a clinical study with an investigational product. Previous participation in this study.

## Screening

Subject eligibility (that subjects fulfill all inclusion criteria and do not meet any exclusion criteria) is established before inclusion, treatment, or randomization.

## Withdrawal criteria

Subjects can discontinue their participation in the study at any time without any consequence to his/her continued treatment. The investigator/sponsor can at any time terminate the study for a subject due to, e.g., unacceptable adverse events/adverse reactions or because the subject does not follow procedures in the study protocol. If the subject discontinues the study, follow-up of this subject will be performed according to the clinic’s routine.

# Study treatments

## Description of investigational product(s)

The study participants will be randomization to either;

1. **Hibitane®** (investigational treatment), manufactured by Bioglan AB, Box 50310, 202 13 Malmö, Sweden. 1 ml contains 10 g chlorhexidine gluconate (1%) and 20 mg Cetostearyl alcohol.

Therapeutic indications: anti-septic use for clinical vaginal examinations, especially during delivery.

Administration time: 3 months with additional treatment in case of infection relapse during the observational phase of the study, 3-6 months after inclusion.

Known adverse event: irritation on skin and mucosa.

Restrictions: allergy to the active substance, avoid contact of active substance with brain or meningeal tissue, or ear canal due to neurotoxicity. Hibitane® is approved for vaginal use even during pregnancy and lactation

or

1. **Fluconazole** (control or standard treatment), manufactured by different producers. Oral capsules of 150 mg fluconazol + 139 mg laktos.

Therapeutic indications: different types of fungal infections (cryptocock meningitis, coccidioido mycosis, invasive candidiasis) including mucosal Candia albicans infections.

Administration time: 3 months with additional treatment in case of infection relapse during the observational phase of the study, 3-6 months after inclusion.

Known adverse events: most common side effects are; headache, abdominal pain, nausea, vomiting, diarrhea, skin rashes. For more information, see attached product resume.

Restrictions: allergy to the active substance, doses should be adjusted in case of reduced renal or liver function, concomitant use of medications with known risk of increasing QT-interval and be metabolized via cytocrom P450 (CYP)3A4 (cisaprid, astemizole, pimozid, quinidine and erythromycin). Fluconazole is not approved during pregnancy. For more information, see attached product resume.

Both medication will be prescribed and handled routinely by the pharmacies.

## Dose and administration

1. Hibitane® is a cream and will be applied vaginally by the help of a 10 ml plastic syringe (Braun, Melsungen, Germany, CE 0123), provided to the participants randomized to Hibitane®. During the first week of treatment 8 ml is used every night at bedtime and thereafter 8 ml will be used at night once a week for 11 consecutive weeks. The dose of 8 ml is considered an adequate to be applied in the vagina for achieving treatment effect. Most of the cream will stay in place, but a small portion will leak and a genital pad is recommended. Dose changes or withdrawal of the medication will be considered in case of AE.
2. Fluconzole® oral 150 mg capsules will be used every 3 days for the first 3 doses, thereafter as prophylactic treatment with 150 mg/week for another 11 consecutive weeks. The fluconazole regime serves as the reference medication normally used for recurrent vulvovaginal candidiasis according to international recommendations and FASS. Fluconazole can be taken regardless of food intake. Dose changes or withdrawal of the medication will be considered in case of AE.

## Packaging, labeling, and handling of investigational products(s)

Both study drugs will be prescribed and marked according to usual practice at the pharmacy. No administration of the drugs will be handled by study personal.

**Hibitane® 1% vaginal cream**

What will be new for the participants is the handling and administration of Hibitane® 1% vaginal cream. The cream comes in a plastic bottle of 250 ml which should be stored at room temperature (< 25° C). After the bottle is opened it can preferable be stored in a fridge between use. Participants randomized to Hibitane® will be provided with 10 ml plastic syringes in surplus for administrations. Participants will be instructed how to insert the cream in the upper part of the vagina. An expire date is marked on each bottle, and the bottle can be stored after it has been opened for at least 3 months according to information from the manufacturer.

A written information of the Hibitane administration is provided.

**Fluconazol®, oral capsules 150 mg**

Most participants are probably already familiar to the fluconazole regime since they suffer from recurrent candida infections. The capsules come in paper packaging of 6 capsules in each. A total of 18 capsules will be prescribed, but additional prescriptions might be needed if the infection relapses. The packaging is marked at the pharmacy and can be kept at room temperature (< 30° C). The capsules should be administered approximately at the same time every day.

A written information of the fluconazole administration is provided.

## Drug accountability and treatment compliance

Compliance will be evaluated at the follow-up visits and the participants will fill out a web based digital weekly dairy how they have taken the study medications. If a dose is missed, it should be administered as soon as possible as stated in the study protocol.

## Randomization

The randomization ratio between the treatment groups will be 1:1. A computer generated block randomization of 15 participants will be performed by the research midwife. Randomization to the study medications will be carried out via opening of opaque sealed envelopes in consecutive order. All women will be identified through a patient log with name and Swedish personal identification number and a study identification number, used on all CRFs (paper and electronic). The randomization will not be blinded to neither participants nor investigators due to the differences in study medications. The primary outcome variable is objective (negative vaginal culture for *Candida albicans*) with low risk of bias.

Subjects are included/randomized consecutively as they are found to be eligible for inclusion in the study. If a subject discontinues their study participation, their subject code will not be reused and the subject will not be allowed to re-enter the study again.

## Blinding

N/A

## Code breaking

There is no blinding and a list for breaking the randomization code is not needed.

## Concomitant medications

Medications that are considered necessary for the safety and well-being of the subject can be given at the discretion of the investigator, unless otherwise specified as an exclusion criterion. Concomitant medications are reported in the CRF.

If treatment fails (positive culture for *Candida albicans* and symptoms), rescue medication of vaginal Clotrimazole 200 mg twice per week will be provided in both study groups for the rest of the study period. The regime of Clomitrazole is another established treatment for vulvovaginal *Candida albicans* infection.

## Destruction

N/A

## Treatment after study end

After the study has ended no further Hibitane® will be prescribed. If the participants have additional need for help with RVVC, they will either be taken care of at the Vulvar Clinic at Danderyd Hospital and provided routine care, or referred to their ordinal open care gynecologist.

# Methods for measurement of endpoints for clinical efficacy and safety

## Methods for measurement of endpoints for clinical efficacy

### Primary endpoint (variable)

The primary endpoint of treatment efficacy is the proportion of women in each group that has negative vaginal cultures for *Candida* albicans after 1 week after active treatment. The cultures will be analyzed at the Microbiology department at Karolinska University Hospital Solna in collaboration with professor Annelie Brauner.

### Secondary endpoints (variables)

1. The secondary endpoint of treatment efficacy is the proportion of women in each group that has negative vaginal cultures for *Candida* albicans at 3 months’ and 6 months’ follow-ups, after end of prophylactic treatment. The cultures will be analyzed as described under 8.1.1.
2. Adverse events will continuously be registered during the study. The participants can fill out any AE in a web based digital weekly diary but also contact the research midwife when needed. AEs will be described and summarized and compared between treatment arms regarding proportion of women with AE.
3. A symptom and examination score (composite index 0-5) will be measured first at baseline/inclusion and then at 3 follow-ups; 1 week after treatment, 3 months’ follow-up after prophylactic treatment and at 6 months’ follow-up. The investigator will ask the participant for common symptoms of RVVC (typical discharge, itching, dryness of the skin/mucosa, burning and pain). Each present symptom will generate 1 point of the composite index (range 0-5). During the gynecological examination performed at inclusion and at each follow-up, typical clinical findings (redness skin/mucosa, typical discharge, dry skin/mucosa, fissures skin/mucosa, visible candida hyfae under the microscope) will be registered in the CRF. Each finding will generate 1 point of the composite index (range 0-5). The proportion of women in each treatment arm with symptom- and examination score >2 for both the will be used for statistical analyses.
4. The content of lactobacillus in vaginal wet mount will be analyzed and semi-quantified first at baseline/inclusion and then at 3 follow-ups; 1 week after treatment, 3 months’ follow-up after prophylactic treatment and at 6 months’ follow-up. Routine procedure for wet mount (vaginal secretion with NaCl) will be performed and the amount of lactobacilli will be graded in a light microscope with high magnification (40x and 100x) (Olympus CX41) as either normal quantity (more lactobacilli compared to number of epithelial cell/view field) or reduced (less or absent lactobacilli compared to number of epithelial cell/view field). Differences in proportion of women with normal/reduced quantity between study groups x time will be analyzed. The two investigators will before the study starts, analyzed a number of wet mounts together for inter-rater reliability. All wet mounts will be photo documented via a camera connected to the microscope placed at the department of obstetrics and gynecology, Danderyd Hospital. These photos will be blinded and evaluated by the two investigators together.
5. Data on events of relapses will be collected during the prophylactic and observational phases of the study. The participants will be provided with equipment to collect self-sampled vaginal cultures for *Candida albicans* at home in case of new symptoms of infection. They can also contact the research midwife who could help out with the cultures if needed. Data on relapses will be reported in the CRF.
6. Biopsies from the vaginal mucosa will be used to study possible toxic effect on the vaginal epithelial cells. The biopsies will be taken at baseline/inclusion and at the 3 months’ follow-up and analyzed by histopathology and immunohistochemistry. The biopsies are 3-4 mm, taken by biopsy forceps after local anesthetics. The purpose of the biopsies is to investigate how CHG affect the vaginal mucosa causing possible toxic reactions of the epithelium and impairment of the local immune response. The biopsies will be microscopically examined using ordinary histopathological staining as well as immunohistochemistry for inflammatory and immunological markers. Also PCR analyses for antimicrobial peptides, cytokines, kemokins, cationic intrinsically disordered antimicrobial peptides (CIDAMP) will be performed. Biopsies from participants allocated to FLZ will serve as controls.
7. The vaginal biopsies described above will also be used to study vaginal biofilm formation before and after treatment and prophylaxis with CHG and FLZ.

All laboratory test will be carried out at Karolinska Institutet core facilities.

The biopsies will be stored in the Stockholm Medical Biobank (registration number 914, sample ID Bb142563).

## Methods for measurement of endpoints (variables) for clinical safety

Two of the secondary endpoints are used for clinical safety;

1. Adverse events will continuously be registered during the study. The participants can fill out any AE in a web based digital diary and also contact the research midwife when needed. AEs will be registered in the CRF. AEs will be analyzed as proportion of women with AE and compared across study arma. For more details, see section 9.
2. Possible toxic effects on the vaginal cells after CHG treatment will be analyzed in vaginal biopsies described under 8.1.2. The analysis will focus on injuries of the epithelial lining and increase of inflammatory and immunological markers (antimicrobial peptides, cytokines, kemokins, CIDAMP). The biopsies will be taken at inclusion and at 3 months’ follow-up, when the prophylactic phase of the study is completed. Biopsies from participants allocated to FLZ will serve as controls.

# Handling of Adverse Events

## Definitions

### Adverse Event (AE)

Adverse Event (AE): Any untoward medical occurrence in a clinical investigation subject administered a medicinal product and, which does not necessarily have a causal relationship with the treatment, can be an unfavorable and unintended sign (including an abnormal laboratory discovery), symptom or disease temporally associated with the use of the medicinal (investigational) product, whether or not related to the medicinal (investigational) product.

### Adverse Reaction (AR)

In the pre-approval clinical experience with a new medicinal product or new use of a medicinal product, and particularly as the therapeutic dose(s) may not be established, all noxious and unintended reactions to the medicinal product related to any dose should be considered an adverse reaction (AR). The phrase “reaction” to a medicinal product means that the causal relationship between the medical product and an adverse event is at least a reasonable possibility, that is the relationship cannot be ruled out.

### Serious Adverse Event (SAE)

Serious adverse event (SAE): Any untoward medical occurrence that at any dose:

- results in death
- is life-threatening
- requires inpatient hospitalization or prolongation of existing hospitalization
- results in persistent or significant disability or incapacity
- results in a congenital anomaly/malformation

Medical and scientific assessment will be made to determine if an event is “serious” and whether it would prompt reporting in other situations, for example important medical events that may not be directly life-threatening or result in death or hospitalization but may compromise the study subject or may require intervention to prevent one of the other results set forth in the definitions above. These should also normally be considered as SAEs.

### Suspected Unexpected Serious Adverse Reaction (SUSAR)

SUSAR: A reaction/event that is unexpected, serious, and suspected to be caused by the treatment, i.e. adverse events that are not included in the Investigator’s Brochure (IB) or SPC.

## Assessment of Adverse Events

### Assessment of causal relationship

The investigator is responsible for determining whether there is a causal relationship between the AE/SAE and use of the investigational product.

Those AEs which are suspected of having a relationship to the investigational product will be followed up until the subject has recovered or is well taken care of and on their way to good recovery (see also section 9.4, Follow-up of Adverse Events).

All AE will be categorized either as likely related, possibly related, or not related, in accordance with the definitions below:

**Likely related**: Clinical event, including abnormal results from laboratory analyses, occurring within a reasonable time after administration of the intervention/investigational product. It is unlikely that the event can be attributed to underlying disease or other medications, but is most likely caused by the investigational product and its emergence is reasonable in relationship with use of the investigational product.

**Possibly related**: Clinical event, including abnormal results from laboratory analyses, occurring within a reasonable time after administration of the intervention/investigational product. The event could be explained by the investigational product and its emergence is reasonable in relationship with use of the investigational product, but there is insufficient information to determine the relationship. The event could be explained by an underlying disease or other medications.

**Not related**: Clinical event, including abnormal results from laboratory analyses, that is not reasonably related to the use of the intervention/investigational product. The event is unlikely related to the intervention/investigational product and can be explained by other medications or underlying disease.

### Assessment of intensity

Each adverse event shall be classified by an investigator as mild, moderate or severe.

**Mild:** The adverse event is relatively tolerable and transient in nature but does not affect the subject’s normal life.

**Moderate**: The adverse event causes deterioration of function but does not affect health. The event can be sufficiently unpleasant and interferes with normal activities but does not completely obstruct them.

**Severe**: The adverse event causes deterioration of function or work ability or poses a health risk to the subject.

### Assessment of seriousness

The investigator is responsible for assessing the seriousness (serious or non-serious). If the incident is considered serious, this should be reported as a serious adverse event (SAE) by the investigator to the sponsor. See also section 9.3.1, Reporting of Serious Adverse Events (SAE).

## Reporting and registration of Adverse Events

At each study visit, adverse events (AE) are continuously registered, starting from start of treatment with the investigational products, up to and including 12 weeks after the subject has ended their treatment with the investigational products. All AE that occur during the study and which are observed by the investigator/study midwife or reported by the subject will be registered in the CRF regardless of whether they are related to the investigational product or not. Assessment of causal relationship, severity, and whether the AE is considered to be an SAE or not will be done by the investigator directly in the *CRF*. The subjects can also report AEs in the digital weekly diary or via communication with the investigator/study midwife. At minimum, for each AE/SAE, a description of the event is recorded (diagnosis/symptom if diagnosis is missing), start and stop dates, causal relationship, severity, if the AE is considered to be an SAE or not, measures and outcome. These data will also be reported in the participant’s medical record.

All reported new symptoms in both study groups will be evaluated as a possible AE.

All AE shall be registered in the CRF from start of treatment to 12 weeks after end of prophylactic treatment *as above (section 9.3, Reporting and registration of Adverse Events)*.

### Reporting of Serious Adverse Events (SAE)

Serious adverse events (SAE) are reported to the sponsor on a special SAE form within 24 hours of the investigator being informed of the SAE.

Follow-up information describing the outcome and handling of the SAE is reported as soon as this information is available. The original should be kept in the Investigator Site File.

### Reporting of Suspected Unexpected Serious Adverse Reactions (SUSAR)

Those SAE which are assessed by sponsor to be SUSAR are reported via a [CIOMS form](https://cioms.ch/wp-content/uploads/2017/05/cioms-form1.pdf) to the European Medicines Agency (EudraVigilance database) according to the specified time frames.

SUSAR that are fatal or life-threatening are reported as soon as possible and no later than 7 days after the incident has become known to the sponsor. Relevant follow-up information is sent thereafter within an additional 8 days. Other SUSAR are reported as soon as possible and no later than 15 days after they have come to the sponsor’s knowledge.

## Follow-up of Adverse Events

Subjects who have been affected by adverse events (AE/SAE) will be followed-up until the adverse event is resolved or stable. Measures taken in case of unacceptable adverse events could be dose adjustment, treatment interruption or withdrawal of from the study.

## Annual Safety Report (Development Safety Update Report, DSUR)

The sponsor will submit an annual safety report to the Swedish Medical Products Agency including a list of all SAE that have occurred as well as possibly SUSAR. A summary assessment of the safety situation for the subjects and a benefit/risk evaluation for the study will also be reported.

## Procedures in case of emergencies, overdose or pregnancy

The sponsor and investigator will immediately take the urgent safety measures to protect the subjects from immediate danger. If needed the study may temporarily be suspended and supplementary monitoring measures might be introduced. The sponsor will inform the Swedish Medical Products Agency and EPM as soon as possible about the urgent safety measures taken.

If a study subject becomes pregnant, this person will be followed up until the birth has taken place. If the fetus/child will have any congenital malformation, this will be reported as a serious adverse event or side effect (SAE).

Adequate contraceptive method is an inclusion criteria and pregnancy test will be checked twice; before start of active and prophylactic treatment.

## Reference Safety Information

SmPC for Fluconazol and Hibitane are attached to the application (attachment 05, 06) The most common expected AE for each study medication are listed in the protocol (7.1).

# Statistics

## Analysis population

The study is a RCT controlled non-inferior trial with 60 women randomly assigned to either vaginal chlorhexidine gluconate (N=30) or oral fluconazole (N=30). Baseline plus 3 follow-up visits (baseline/inclusion, 1 week, 3 months and 6 months) are planned.

## Statistical analyses

### Statistical methods

***Objective:*** The aim of this study is to analyze whether vaginal chlorhexidine gluconate (CHG) is at least as effective and safe as oral fluconazole (FLZ) as treatment and prophylaxis for recurrent vulvovaginal candidiasis.

***Study design:*** Randomized controlled non-inferior trial with 60 women randomly assigned to either vaginal chlorhexidine gluconate (N=30) or oral fluconazole (N=30). Baseline plus 3 follow-up visits (baseline/inclusion, 1 week, 3 months and 6 months) are planned.

***Primary outcome****:* Proportion of women with negative cultures for *Candida albicans* 1week post-treatment.

***Secondary outcomes****:*

- Negative cultures for *Candida albicans* at 3 months’ and 6 months’ follow-ups, after end of prophylactic treatment.
- Analysis of proportion of women with adverse events (AE). The AEs will also be reported in descriptive terms and summarized for each treatment arm.
- Proportion of women with symptom score > 2. Each symptom of typical discharge, itching, dryness of the skin/mucosa, burning and pain will generate 1 point of a composite index (range 0-5).
- Proportion of women with examination score >2. Each finding of redness skin/mucosa, typical discharge, dry skin/mucosa, fissures skin/mucosa, visible candida hyphae in the microscope will generate 1 point of a composite index (range 0-5).
- Proportion of women with reduced vaginal lactobacilli content in vaginal smears measured by a semi-quantitative method as normal or reduced quantity.
- Proportion of women with relapse of *Candida albicans* infection between end of treatment and end of prophylactic treatment (1week post treatment to 3 months’ follow-up) and between 3- and 6 months’ follow-up after end of prophylactic treatment.
- Proportion of women with possible toxic effect on the vaginal epithelium, defined as disruption and/or inflammation of the epithelial lining, analyzed from vaginal biopsies before and after treatment and prophylaxis.
- Further analysis of mucosa inflammatory mechanism and biofilm formation during and after treatment and prophylaxis, analyzed from vaginal biopsies. The methods for these *in vitro* exploratory analyses are not yet decided on.

***Statistical methods:***

Analysis for differences over time for treatment efficacy, AEs, symptom- and examination score, impact on vaginal lactobacilli and relapses will be analyzed for all randomized patients (i.e. intent-to-treat ITT). Additionally, we will also examine efficacy for the per-protocol (PP) population. Four time points will be used to study the efficacy (proportion of participants with negative cultures for *Candida albicans*) in each treatment arm (baseline, 1w post-treatment, follow-up 3 and 6 months). We will examine the difference in means, proportions and median values using 95% confidence intervals (CI). The CI interval will be computed using 1) for a proportion in one sample and 2) logistic or multinomial logistic regression analysis.

Differences in baseline characteristics between the CHG and FLZ groups will be analyzed with Chi2 for categorical data, Mann Whitney U test for ordinal data, or t-test for continuous data to examine potential selection bias at randomization. Differences in drop-out rates across treatment arms will also be analyzed using Mann Whitney U test.

### Drop-outs

Differences in drop-out rates across treatment arms and baseline characteristics will be analyzed using Mann Whitney U test. Efficacy and safety will be analyzed for all randomized patients (i.e. intent-to-treat ITT). Additionally, we will also examine efficacy for the per-protocol (PP) population. We will examine the difference in dropout rates and missing visits across treatment arms.

## Adjustment of significance and confidence interval

For the secondary outcomes, we adjust for the rate of Type I errors by adjusting the p-values by controlling for the false discovery rate (the proportion of Type I errors in all significant findings) among all secondary outcomes examined.

## Sample size calculations

The sample size and calculated power for the primary outcome of negative cultures for *Candida albicans* is based on data in Alvendal et al. Acta Obstet Gynecol Scand. 2017. In that study there was 100% positive cultures for *Candida albicans* before routine treatment with fluconazole for RVVC. After fluconazole treatment, 14% participants were still culture positive, with 86% success rate of primary outcome. Using the presumption: α= 5% β= 90%, success for routine FLZ treatment= 86%, success for experimental treatment with CHG in the current study= 86% with non-inferiority limit= 30%, 46 participants are needed (23 in each group) to achieve a power of 90 % not to exclude an effect-size of 30%. 60 participants will be included in case of drop-outs. All test will be performed at the 5% significance level.

## Interim analysis (if relevant)

No interim analysis will be performed.

# Quality Control and Quality Assurance

The sponsor is responsible for planning the Quality Control and Assurance with regular monitoring. An independent monitor has been assigned and will perform the monitoring as describe 11.2.

## Quality Assurance and Sponsor oversight

The clinical trial will be performed at the research department at the Department of OB&GYN, Danderyd Hospital. The sponsor has the responsibility for the monitoring plan and to assure direct access to all source data for monitoring, audits and inspections.

Only 3 personnel will be involved in the study; the principal- and co-investigator together with the research coordinator. The study personnel have long experience of working together and have planned the study in close co-operation. No special training of personnel is needed.

The study coordinator role is to book the appointments for the participants, collect data from vaginal cultures and other laboratory tests, handle the CRFs and be of help during monitoring and audits. Any AEs can directly be reported to the study coordinator and further reported to the investigators and sponsor if needed.

The principal (=sponsor) and co-investigator will conduct the study visits, give the oral and written information and collect the signed consents, check the inclusion- and exclusion criteria and decide on the final inclusion of the participants.

The major risk of the study has been identified to be correlated to the study drug fluconazole which serves as the reference treatment in the trial. Fluconazole is an approved medication and caution to avoid known AEs has been taken in the inclusion- and exclusion criteria in the protocol. No serious AEs with Hibitane is expected. Any AEs can be reported directly to the study coordinator or by the use of the electronic weekly dairy.

## Monitoring

The study will be monitored by an independent monitor before the study begins, during the study conduct, and after the study has been completed, so as to ensure that the study is carried out according to the protocol and that data is collected, documented, and reported according to ICH-GCP and applicable ethical and regulatory requirements. Monitoring is performed as per the study’s monitoring plan and is intended to ensure that the subject’s rights, safety, and well-being are met as well as data in the CRF are complete, correct, and consistent with the source data.

## Source data

The source data is listed below. The monitor will have access to all source data and subjects have provided consent by signing the Subject Information and Informed Consent.

**Source data list**

eCRF Web based questionnaire of medical history and reproductive health

Web based weekly dairy

CRP (paper) Signed oral and written informed consent

Inclusion and exclusion

Data from each visit is directly registered in the CRF

Medical records All visits will be registered in the participant’s medical record (TakeCare). Any AE or other clinical deviations will be reported in the medical records.

## Deviations or serious breaches

Serious breaches and deviations from the study protocol, GCP and other regulations that significantly and directly affects, or with high likelihood could affect, the subjects in Sweden or the scientific value of the study, shall be immediately reported within 7 days (from knowledge) to the Swedish Medical Products Agency. It is the sponsor’s responsibility to judge the consequences of deviations that have occurred, and thus also to decide whether the Swedish Medical Products Agency should be informed.

Minor deviations that do not affect subjects’ integrity or safety, nor significantly affect the study’s scientific value, are documented in the study documentation of the principal investigator and the sponsor.

## Audits and inspections

Authorized representatives for the sponsor and Competent Authorities (CA) may carry out audits or inspections at the study site, including source data verification. The investigator will ensure that all source documents are available for audits and inspections. The purpose of an audit or inspection is to systematically and independently review all study-related activities and documents, so as to determine whether these activities were performed, registered, analyzed and reported correctly according to protocol, Good Clinical Practice (GCP) and applicable regulations.

# Ethics

## Compliance to the protocol, GCP and regulations

The study will be performed in compliance with the study protocol, the Declaration of Helsinki, ICH-GCP (Good Clinical Practice) guidelines and current national and international regulations governing this clinical trial. This is to ensure the safety and integrity of the study subjects as well as the quality of the data collected.

## Ethical review of the study

The final study protocol for clinical trials must be approved, as a part of the application for a permit for clinical trials, by both the Swedish Ethical Review Authority (Etikprövningsmyndigheten, EPM) and the Swedish Medical Products Agency before the trial can be conducted. The final version of the informed consent form and other information provided to subjects, must be approved or given a written positive opinion by EPM. EPM and the Swedish Medical Products Agency must be informed of any changes in the study protocol in accordance with current requirements. *See also section 13, Substantial changes to the study.*

## Procedure for obtaining informed consent

The principal investigator at each site shall ensure that the subject is given full and adequate oral and written information about the study, its purpose, any risks and benefits as well as inclusion and exclusion criteria. Subjects must also be informed that they are free to discontinue their participation in the study at any time without having to provide a reason. Subjects should be given the opportunity to ask questions and be allowed time to consider the provided information. If the person chooses to participate, both the subject and the investigator shall sign the informed consent form. A copy of the subject information as well as the informed consent form shall be provided to the subject. The subject’s signed and dated informed consent must be obtained before performing any study-specific activity in the study. Each subject who participated in the study will be identified by a subject number on a subject identification list. The subject agrees that monitors, auditors, and inspectors may have access to their medical records and other source data. If new information is added to the study, the subject has the right to reconsider whether he/she will continue their participation.

## Data protection

If any part of the data is handled by any other organization, inside or outside the EU, appropriate agreements and/or other documentation will be established, to ensure that the data processing is performed in accordance with the provisions of the General Data Protection Regulation (GDPR) and other relevant legislation, before any data transfer takes place.

The content of the informed consent form complies with relevant integrity and data protection legislation. In the subject information and the informed consent form, the subject will be given complete information about how collection, use and publication of their study data will take place. The subject information and the informed consent form will explain how study data are stored to maintain confidentiality in accordance with national data legislation. All information processed by the sponsor will be pseudonymized and identified with a study code.

The informed consent form will also explain that for verification of the data, authorized representatives of the sponsor, as well as relevant authority, may require access to parts of medical records or study records that are relevant to the study, including the subject’s medical history.

## Insurances

All participants are insured through Swedish patient insurance and the Swedish patient injury act (Patientskadelagen (1996:799)). Both investigational products (Hibitane°® and Fluconazol® HEXAL) are included in the Swedish Pharmaceutical Insurance (Läkemedelsförsäkring).

# Substantial changes to the study

Substantial changes to the signed study protocol are only possible through approved protocol amendments and by agreement from all responsible persons. Information on non-substantial changes should be clearly noted in the amended protocol.

In the event that substantial changes to the protocol (e.g., changing of the main objective, primary or secondary variables, method to measure the primary variable, changing of the investigational product or dosage) will be made during the course of the study, approval from the Swedish Ethical Review Authority (Etikprövningsmyndigheten, EPM) as well as the Swedish Medical Products Agency (Läkemedelsverket) shall be obtained before any changes are implemented. A change that concerns a new site, new investigator or a new study patient information sheet shall only be approved by EPM.

Non-substantial changes will be recorded and later entered in documentation that is submitted, for example in any subsequent notifications of a substantial change or in connection with End of Trial reporting.

# Collection, handling, and archiving data

Subjects who participate in the study are coded with a specific study identification number. All subjects are registered in a subject identification list (subject enrolment and identification list) that connects the subject’s name and personal number with a study identification number.

All data will be registered, managed, and stored in a manner that enables correct reporting, interpretation, and verification. The complete Trial Master File, as well as source documents, will be archived for at least 10 years after the study is completed. Source data in the medical records system is stored and archived in accordance with the respective hospital regulations.

## Case Report Form (Forskningspersonsformulär)

A paper Case Report Form (CRF) is used for data collected at all five study visits. An electronic CRF (Entermedic) will be used to collect some data; 1) the questionnaire on medical history and reproductive health, filled out at inclusion, 2) a weekly diary where the participants can fill out compliance to the allocated treatment, symptoms of RVVC and AEs.

The investigator must ensure that data is registered and any corrections in the CRF are made as stated in the study protocol and in accordance with the instructions. The investigator must ensure that the registered data is correct, complete, and that reporting takes place according to the timelines that have been predefined and agreed. The investigator signs the completed CRF. A copy of the completed CRF will be archived at the study site.

If an examination/test is not performed and data does not exist, ND (Not done) or NK (Not known) is marked. If the question is irrelevant NA (Not applicable) is written. Corrections in the paper CRF are done by striking out the incorrect information and adding the correct information next to the incorrect information, signing, and dating the correction.

Each study visit will also be registered in the hospital’s medical records.

# Notification of study completion, reporting, and publication

The Swedish Medical Products Agency shall be informed of the study’s completion at latest 90 days after study end, through submission of a ”Declaration of End of Trial Notification” form.

Within one year after the study is completed, the results shall be analyzed, a clinical study report with individual data shall be prepared, and the study results shall also be reported in the EudraCT database.

# References

1. Sobel JD. Vulvovaginal candidosis. Lancet. 2007;369(9577):1961-71.

2. Fidel PL, Jr. History and update on host defense against vaginal candidiasis. Am J Reprod Immunol. 2007;57(1):2-12.

3. Sobel JD. Recurrent vulvovaginal candidiasis. American journal of obstetrics and gynecology. 2016;214(1):15.

4. Farmer MA, Taylor A, Bailey AL, Tuttle A, Macintyre LC, Milagrosa Z, et al. Repeated Vulvovaginal Fungal Infections Cause Persistent Pain in a Mouse Model of Vulvodynia. Science Translational Medicine. 2011;3(101).

5. Sobel JD, Wiesenfeld HC, Martens M, Danna P, Hooton TM, Rompalo A, et al. Maintenance fluconazole therapy for recurrent vulvovaginal candidiasis. The New England journal of medicine. 2004;351(9):876-83.

6. Rosa MI, Silva BR, Pires PS, Silva FR, Silva NC, Souza SL, et al. Weekly fluconazole therapy for recurrent vulvovaginal candidiasis: a systematic review and meta-analysis. European Journal of Obstetrics and Gynecology. 2013;167(2):132-6.

7.Marchaim D, Lemanek L, Bheemreddy S, Kaye KS, Sobel JD. Fluconazole-resistant Candida albicans vulvovaginitis. Obstetrics and gynecology. 2012;120(6):1407-14.

8.FASS. 8 ed. ed: Oxford University Press; 2010.

9. Alvendal C, Mohanty S, Bohm-Starke N, Brauner A. Anti-biofilm activity of Chlorhexidine gluconate against Candida albicans vaginal isolates. PloS one. 2020;15(9):e0238428.

10. Kumar A, Alam A, Rani M, Ehtesham NZ, Hasnain SE. Biofilms: Survival and defense strategy for pathogens. International Journal of Medical Microbiology. 2017;307(8):481-9.

11. Al-Niaimi A, Rice LW, Shitanshu U, Garvens B, Fitzgerald M, Zerbel S, et al. Safety and tolerability of chlorhexidine gluconate (2%) as a vaginal operative preparation in patients undergoing gynecologic surgery. AJIC: American Journal of Infection Control. 2016;44(9):996-8.

12. Shubair M, Stanek R, White S, Larsen B. Effects of Chlorhexidine Gluconate Douche on Normal Vaginal Flora. Gynecologic and Obstetric Investigation. 1992;34(4):229-33.

13. Patton LD, Sweeney CY, Rabe KL, Hillier LS. The Vaginal Microflora of Pig-Tailed Macaques and the Effects of Chlorhexidine and Benzalkonium on This Ecosystem. Sexually Transmitted Diseases. 1996;23(6):489-93.

# Attachments

SAP (Statistical Analysis Plan)
