# Supplementary material for: Vaginal chlorhexidine gluconate versus fluconazole for recurrent vulvovaginal candidiasis: A randomized noninferiority trial
Source: PLoS One. 2026 Jan 20;21(1):e0340862. doi: 10.1371/journal.pone.0340862 (PMC12818662; doi:10.1371/journal.pone.0340862)
Supplement: S2 File — (DOCX) [file pone.0340862.s004.docx]

**Checklist of Items for Reporting Noninferiority or Equivalence Trials (Additions or Modifications to the CONSORT Checklist are Shown in Italics)**

[**https://www.equator-network.org/reporting-guidelines/consort-non-inferiority/**](https://www.equator-network.org/reporting-guidelines/consort-non-inferiority/)

| **Paper Section and Topic** | **Descriptor (Adapted for Noninferiority or Equivalence Trials)** * | **Reported on page No** |
| --- | --- | --- |
| Title and abstract | How participants were allocated to interventions (eg, “random allocation,” “randomized,”  or “randomly assigned”), *specifying that the trial is a noninferiority or equivalence trial.* | Title page  Abstract - methods |
| **Introduction**  Background | Scientific background and explanation of rationale, *including the rationale for using a*  *noninferiority or equivalence design*. | Introduction, p 4, lines 86-95 |
| **Methods** |  |  |
| Participants | Eligibility criteria for participants *(detailing whether participants in the noninferiority or*  *equivalence trial are similar to those in any trial[s] that established efficacy of the*  *reference treatment)* and the settings and locations where the data were collected. | P 5, liner 120-126 |
| Interventions | Precise details of the interventions intended for each group, *detailing whether the*  *reference treatment in the noninferiority or equivalence trial is identical (or very similar)*  *to that in any trial(s) that established efficacy,* and how and when they were actually  administered. | P 6, lines 139-143 |
| Objectives | Specific objectives and hypotheses, *including the hypothesis concerning noninferiority or*  *equivalence.* | P 4, lines 99-103 |
| Outcomes | Clearly defined primary and secondary outcome measures, *detailing whether the*  *outcomes in the noninferiority or equivalence trial are identical (or very similar) to*  *those in any trial(s) that established efficacy of the reference treatment* and, when  applicable, any methods used to enhance the quality of measurements (eg, multiple  observations, training of assessors). | P 5, lines 111-119 |
| **Sample size** |  | Statistical Analyses, p 10-11, line 232-240 |
| **Randomization** |  |  |
| Sequence generation | Method used to generate the random allocation sequence, including details of any  restriction (eg, blocking, stratification). | P 6, lines 144-150 |
| Allocation concealment | Method used to implement the random allocation sequence (eg, numbered containers or central telephone), clarifying whether the sequence was concealed until interventions  were assigned. | P 6, lines 145-147 |
| Implementation | Who generated the allocation sequence, who enrolled participants, and who assigned  participants to their groups. | P 6, lines 144-147 |
| **Blinding (masking)** | Whether or not participants, those administering the interventions, and those assessing the outcomes were blinded to group assignment. When relevant, how the success of blinding was evaluated. | P 6, lines148-150 |
| **Statistical methods** |  | Statistics p3 |
| **Results** |  |  |
| Participant flow | Flow of participants through each stage (a diagram is strongly recommended).  Specifically, for each group report the numbers of participants randomly assigned,  receiving intended treatment, completing the trial protocol, and analyzed for the  primary outcome. Describe protocol deviations from trial as planned, together with  reasons. | Fig.1 |
| Recruitment | Dates defining the periods of recruitment and follow-up. | P 11, lines 249-250 |
| Baseline data | Baseline demographic and clinical characteristics of each group. | Table 1 |
| Numbers analyzed | Number of participants (denominator) in each group included in each analysis and  whether *“intention-to-treat” and/or alternative analyses were conducted*. State the  results in absolute numbers when feasible (eg, 10/20, not 50%). | P 13, lines 250-253  Intention-to-treat is N/A, due to limited number of participants |
| Outcomes and estimation | For each primary and secondary outcome, a summary of results for each group and the  estimated effect size and its precision (eg, 95% confidence interval). *For the*  *outcome(s) for which noninferiority or equivalence is hypothesized, a figure showing*  *confidence intervals and margins of equivalence may be useful.* | P 14-18, Table 2. Effect size was not possible to estimate due to limited number pf participants. |
| Ancillary analyses | Address multiplicity by reporting any other analyses performed, including subgroup  analyses and adjusted analyses, indicating those prespecified and those exploratory | N/A |
| Adverse events | All important adverse events or side effects in each intervention group. | Table S1 |
| **Discussion** |  |  |
| Interpretation | Interpretation of the results, taking into account the *noninferiority or equivalence*  *hypothesis and any other* trial hypotheses, sources of potential bias or imprecision  and the dangers associated with multiplicity of analyses and outcomes. | P 20-22 |
| Generalizability | Generalizability (external validity) of the trial findings. | P 20-22 |
| Overall evidence | General interpretation of the results in the context of current evidence. | P 22, lines 423-433 |
